# Patchy Membrane-Directed Multiphase Complex Coacervation

**DOI:** 10.1021/jacs.6c11826

**Published:** 2026-07-27

**Authors:** Siwen Sun, Nadia Erkamp, Sander G. A. M. Huisman, Alexander B. Cook, Arjan Hazegh Nikroo, Zhuping Yin, Jianhong Wang, Madelief A. M. Verwiel, Jan C. M. van Hest

**Affiliations:** Bio-Organic Chemistry, Departments of Biomedical Engineering and Chemical Engineering and Chemistry, Institute for Complex Molecular Systems, 3169 Eindhoven University of Technology, 5600 MB Eindhoven, The Netherlands

## Abstract

Multiphase separation is a key feature in biomolecular condensates to regulate biological function. In cells, phase separation can occur in a bulk solution or on a lipid membrane. To establish life-like features in artificial cells, much research has been carried out to reproduce multiphase separation behavior. However, this has been mostly restricted to bulk properties, whereas membrane interactions are much less investigated. Here, we introduce a membrane-based strategy in which interfacial electrostatic heterogeneity governs multiphase separation in amylose-based coacervates, enabling the formation of subcompartments localized near the membrane interface. By integrating bowl-shaped polymer vesicles (stomatocytes) decorated with negatively charged gold nanoparticles into a terpolymer membrane, we construct a patchy membrane architecture that introduces localized, highly charged interfacial domains, thereby stabilizing amylose-based coacervates. Upon the addition of succinylated bovine serum albumin as a second phase-forming component, the embedded stomatocytes allow precise control over multiphase separation. Modulating the stomatocytes’ coverage at the coacervate interface enables systematic tuning of the size, spatial localization, and number of protein-enriched subdroplets. Complete stomatocyte coverage further enhances multiphase stability against variations in ionic strength and pH, while improving long-term storage stability. Moreover, the interfacial multiphase organization can be dynamically regulated by ionic strength and temperature. Furthermore, the enlarged interfacial area promotes enhanced cargo uptake, while the programmable positioning of enzyme-containing subcompartments enables spatial regulation of interprotocellular chemical communication. Together, these findings establish interfacial electrostatic heterogeneity as a versatile strategy for engineering membrane-associated multiphase protocells in which spatial organization governs molecular transport, catalysis, and communication.

## Graphical abstract

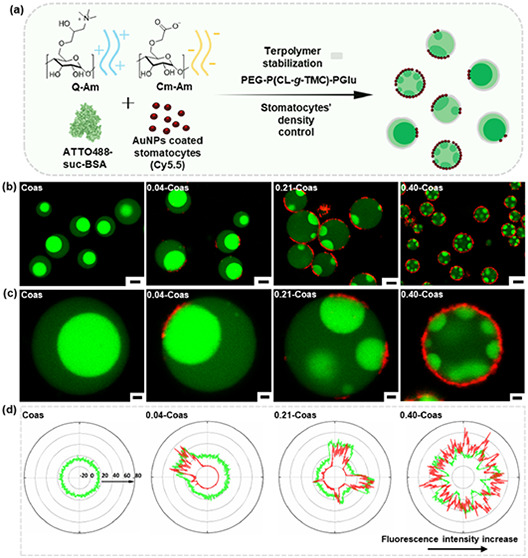

## Introduction

Living cells rely on phase separation as a fundamental mechanism to compartmentalize biomolecules.^1–3^ Their dynamic organization into dense, membraneless condensates facilitates key cellular functions such as signaling,^4–6^ metabolism,^7–9^ and gene regulation.^10–12^ Importantly, cellular liquid–liquid phase separation often results in multiphase coacervates composed of coexisting liquid compartments with distinct compositions and material properties.^13^ Representative examples include the layered organization of the nucleolus,^14^ as well as multiphase or subcompartmentalized structures in stress granules,^15^ P granules,^16^ and nuclear speckles,^17^ highlighting how phase hierarchy underpins functional compartmentalization in cells. Besides multiphase separation in the bulk condensate phase, also interactions of proteins with biological membranes induce phase separation, leading to the assembly of biomolecular condensates near or even tethered to membranes.^18–20^ Key factors such as membrane curvature, lipid composition, and protein–lipid interactions are crucial for the formation of these phase-separated regions at cellular membranes.^19,21^ In many reconstituted and biological systems, membrane binding significantly enhances the propensity for phase separation of proteins and leads to the formation of membrane-associated condensates.^22–26^ Certain intrinsically disordered proteins can form membrane-associated condensates, in which the liquid-like droplets are tethered to the membrane via electrostatic interactions between positively charged protein domains and negatively charged lipids such as phosphatidylserine or phosphatidylinositol 4,5-bisphosphate.^27^ Several protein condensates are formed through protein–protein interactions and are preferentially enriched at membrane surfaces, as exemplified by synapsin-1 condensates.^28,29^ These condensates retain liquid-like properties, including fluidity and fusion capability, while their spatial localization and assembly are modulated by membrane properties such as lipid composition and surface charge.

Coacervates are typically formed by the spontaneous phase separation of oppositely charged polyelectrolytes or other macromolecules, creating liquid–liquid phase separation droplets that can encapsulate proteins,^30,31^ nucleic acids^32,33^ and small molecules.^34^ This feature makes coacervates an excellent platform for artificial cell research, studying the principles of cellular compartmentalization and exploring the behavior of biomolecules in confined environments.^35–40^ Similar to cellular condensates, coacervates can also exhibit multiphase separation.^41–45^ Lu et al. reported that mixtures of electrostatically driven phase-separated systems with distinct critical salt concentrations spontaneously organize into multiphase coacervate droplets.^41^ Zhou et al. developed a dual-stimulus programmed coacervate system that enables sequential pH- and salt-triggered transitions from membrane-less coacervates to nested multiphase coacervates and, ultimately, vesicular-like multiphase architectures.^46^ In our group, we demonstrated that coacervates composed of two polyanionic species and a polycation could be triggered using a kinetic process into a multiphase separated architecture, which was further stabilized via a cross-linking process.^47,48^ Despite the significance of membrane-anchored phase separation in natural systems, research on multiphase separation near or even tethered to membranes in artificial systems, such as coacervates, remains limited.^18,49^ Recent studies have shown that segregative phase separation can regulate condensates within lipid-bounded synthetic cells, illustrating multiphase separation in close association with membranes.^50^ However, the limited number of such examples largely stems from the fact that most existing artificial membrane systems are designed to be uniform, focusing primarily on properties such as stability, fluidity or permeability. In contrast, heterogeneous membranes—with spatial variations in composition, curvature, or mechanical properties—offer a unique opportunity to exert more precise control over the positioning and organization of internal droplets. By introducing controlled heterogeneities, it becomes possible to guide multiphase assemblies to specific membrane regions, regulate droplet size and coalescence, and even mimic the spatial organization observed in natural cellular compartments. However, engineering such heterogeneity in synthetic membranes poses significant technical challenges, including stable patterning at the nanoscale, maintaining membrane integrity, and ensuring compatibility with the dynamic behavior of coacervates or other phase-separated entities.

In this work, we introduce a novel concept of controllable multiphase separation at the membrane interface, governed by patchy membranes. Previously, we showed that the integration in a terpolymer membrane of negatively charged bowl-shaped polymer vesicles, or stomatocytes, decorated with gold nanoparticles (AuNPs), stabilized coacervates composed of oppositely charged amylose derivatives, thereby forming a patchy membrane.^51,52^ Here, we demonstrate that the membrane’s patchiness triggers multiphase separation upon the introduction of a second negatively charged coacervate component, succinylated bovine serum albumin (suc-BSA). By modulating the density of stomatocytes at the coacervate interface, we could precisely control the position, size, and number of BSA-enriched subdroplets within the terpolymer-stabilized coacervates. Moreover, this system allowed for dynamic control through variations in ionic strength and temperature. As an application, the patchy membrane-guided multiphase architecture provides a platform for spatially regulated and interfacially programmed chemical reactions within protocell-like compartments.

## Results and Discussion

### Interface-Anchored Controlled Multiphase Separation

Building on our previous work, poly­(ethylene glycol)-poly­(d,l-lactic acid) (PEG–PDLLA) block copolymers (PEG_22_–PDLLA_95_ and PEG_44_–PDLLA_95_) were self-assembled into polymersomes, which subsequently underwent dialysis-induced shape transformation to yield bowl-shaped stomatocytes.^53^ Upon decoration with gold nanoparticles (AuNPs), the resulting stomatocytes exhibited pronounced interfacial affinity toward net-positively charged amylose-based coacervates, leading to enhanced coacervate stability (Figure S1).^51,52^ We furthermore demonstrated that combining the membrane-forming terpolymer poly­(ethylene glycol)-*b*-poly­(ε-caprolactone-*g*-trimethylene carbonate)-*b*-poly­(glutamic acid) (PEG_44_-*b*-P­(CL_53_-*g*-TMC_54_)-*b*-PGlu_11_) with stomatocytes to stabilize the coacervates yielded a patchy membrane with a tunable nanoparticle density, in which the particles were firmly embedded within the terpolymer matrix (Figure S2).^52^ We now expand this system by introducing an additional negatively charged coacervate component, succinylated bovine serum albumin (suc-BSA), into the system (Figure 1a).

**Figure 1:**
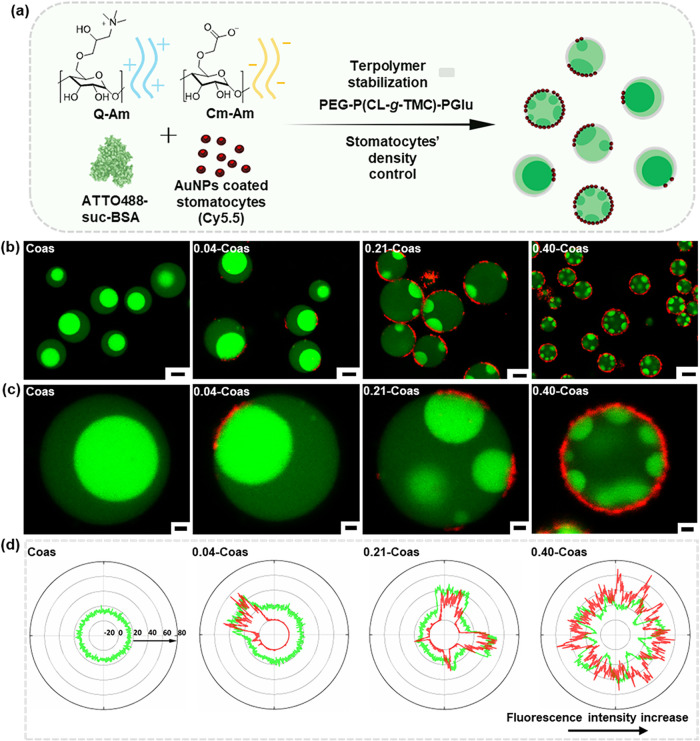
(a) Schematic illustration of a patchy membrane composed of stomatocytes and terpolymer, inducing multiphase separation within coacervates composed of succinylated BSA and charged amylose derivatives. (b and c) CLSM images of suc-BSA-loaded coacervates with different stomatocyte concentrations (0.04-Coas, 0.21-Coas, and 0.40-Coas). Composition: negatively charged amylose (Cm-Am; 33 μL, 1 mg/mL), positively charged amylose (Q-Am; 66 μL, 1 mg/mL), suc-BSA (5 μL, 3.33 mg/mL), stomatocytes (0, 1, 5, or 10 μL, 4.76 mg/mL), and terpolymer (5 μL, 25 mg/mL in PEG 350) in phosphate-buffered saline (PBS). Coacervates with different bulk stomatocyte concentrations are designated as 0.04-Coas (0.04 mg/mL stomatocytes), 0.21-Coas (0.21 mg/mL stomatocytes), and 0.40-Coas (0.40 mg/mL stomatocytes). Red: stomatocytes loaded with Cy5.5, green: ATTO-488 conjugated suc-BSA. Scale bars represent 5 μm (b) and 1 μm (c). (d) Fluorescence intensity line profiles along the perimeter of suc-BSA-loaded 0.04-Coas, 0.21-Coas, and 0.40-Coas droplets. The black arrow indicates a gradual increase in fluorescence intensity. The red line corresponds to stomatocytes (Cy5.5), and the green line corresponds to suc-BSA (ATTO 488). Panels b and c were processed using identical brightness and contrast settings to ensure comparability within the group. All images in panels b and c are shown for visualization purposes only and are not used for quantitative fluorescence analysis. The line profile analysis in panel d was performed on the raw, unprocessed images corresponding to those presented in panel c.

Coacervation was initiated by mixing negatively charged amylose (carboxymethyl-modified, Cm-Am; 33 μL, 1 mg/mL), suc-BSA (5 μL, 3.33 mg/mL), stomatocytes (0, 1, 5, or 10 μL, 4.76 mg/mL), and positively charged amylose (quaternary amine-modified, Q-Am; 66 μL, 1 mg/mL) in phosphate-buffered saline (PBS). After vigorous shaking for 4 min, the terpolymer was added. The terpolymer spontaneously assembled on the surface of the nascent coacervate droplets, thereby stabilizing them against coalescence. The resulting BSA-loaded, terpolymer-stabilized coacervates with varying bulk stomatocyte concentrations were labeled as 0.04-Coas, 0.21-Coas, and 0.40-Coas, respectively (Figure 1b).

Interestingly, membrane-associated multiphase separation emerged within these coacervate droplets. BSA-enriched subdroplets were confined to the inner interfacial zone of coacervates, precisely where the interfacial membrane was decorated by a patch of stomatocytes (Figure 1c). For Coas and 0.04-Coas droplets, a single BSA-enriched subcompartment resided within each coacervate droplet. However, as the number of stomatocytes decorating the coacervate interface increased, the number of BSA-enriched subdroplets concomitantly increased (Figure 1b). For 0.21-Coas droplets, multiple BSA-enriched subdroplets were routinely detected. For 0.40-Coas droplets, more and smaller BSA-enriched subdroplets were observed within each coacervate droplet. As shown in Figure S3, increasing the concentration of stomatocytes at the coacervate interface—from Coas to 0.04-Coas, 0.21-Coas, and 0.40-Coas—correlates with a progressive increase in the number of BSA-enriched subdroplets formed within the interior. To further investigate the spatial relationship between stomatocytes and BSA-enriched subdroplets, fluorescence intensity line profiles were extracted along the perimeter of representative coacervate droplets (Coas, 0.04-Coas, 0.21-Coas, and 0.40-Coas). In these profiles, the red channel corresponded to stomatocytes, while the green channel corresponded to BSA (Figure 1d). Consistent with 2D confocal laser scanning microscopy (CLSM) images, the line profile analysis revealed a stochastic and anisotropic distribution of stomatocytes at the coacervate interface, particularly in 0.04-Coas and 0.21-Coas. Notably, the red signal (stomatocytes) and green signals (BSA) overlapped at their peaks, indicating a positional correspondence between the internal BSA-enriched subdroplets and the stomatocytes at the interface of coacervates. The slight imperfection in spatial correlation between the red and green signals—particularly in 0.21-Coas—arises from the limitation of performing fluorescence analysis in the *x*–*y* plane at a fixed z-position. In the 0.40-Coas system, suc-BSA-enriched droplets are spatially separated to prevent fusion, whereas stomatocytes form an almost complete interfacial coverage. The mismatch in 2D images reflects this packing constraint rather than actual spatial exclusion.

Having examined the spatial organization of stomatocytes and BSA-enriched subdroplets within a single *x*–*y* plane, we next analyzed their relative positioning across multiple *x*–*y* planes along the *z*-axis (Figure 2a). Along the *z*-axis, we observed that BSA-enriched subdroplets within the coacervates exhibited a strong spatial correspondence with stomatocytes localized at the interface across different *x*–*y* planes. The 3D z-stack figures further supported these observations by providing complementary top-view and side-view perspectives (Figure 2b). At the interface where internal BSA-enriched subdroplets formed, stomatocytes often exhibited a relatively continuous, patch-like distribution. For stomatocytes that were sparsely distributed along the interface—particularly in 0.04-Coas and 0.21-Coas—we found that some of these isolated nanoparticles were associated with the formation of smaller BSA-enriched subdroplets directly beneath them, whereas others did not show any apparent association with a corresponding subdroplet within the interior (Figure S4). Three representative droplets were selected for each condition (0.04-Coas, 0.21-Coas, and 0.40-Coas), and the number of visually identifiable subdroplets was accurately determined from the z-stack images. This analysis further confirmed that an increase in the density of stomatocytes decorating the coacervate interface was accompanied by a corresponding increase in the number of internal subdroplets (Figure 2c).

**Figure 2:**
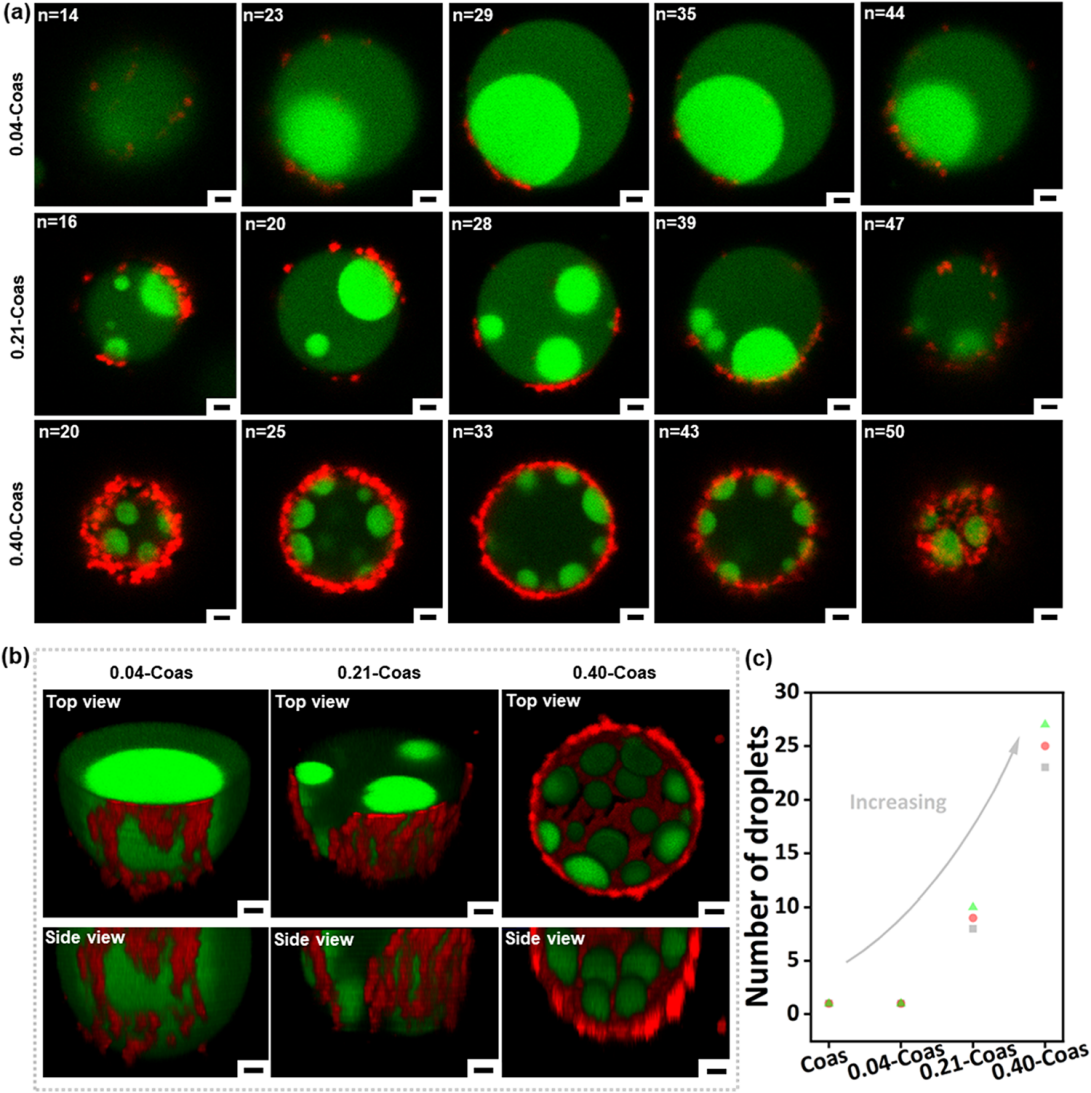
(a) Confocal z-stack micrographs acquired along the *z*-axis for representative BSA-loaded 0.04-Coas, 0.21-Coas, and 0.40-Coas droplets. For each droplet, a total of 60 optical slices were acquired from the bottom to the top (or vice versa). The number *n* denotes the *n*-th optical slice counted from the starting plane (bottom or top) of the droplet. (b) Corresponding 3D z-stack micrographs of the droplets shown in panel (a), presented as top-view and side-view projections. (c) Quantification of the exact number of BSA-enriched subdroplets within individual coacervates for three representative droplets per condition (Coas, 0.04-Coas, 0.21-Coas, and 0.40-Coas), extracted from confocal z-stack images. Red: stomatocytes loaded with Cy5.5, green: ATTO-488 conjugated suc-BSA. Scale bars represent 1 μm.

We subsequently investigated subdroplet positioning in membrane-free coacervates to distinguish the effect of the stomatocytes and the membrane polymer on this process. Coacervation was initiated by combining negatively charged Cm-Am (33 μL, 1 mg/mL), stomatocytes (0, 1, 5, or 10 μL; 4.76 mg/mL), suc-BSA (5 μL, 3.33 mg/mL), and positively charged Q-Am (66 μL, 1 mg/mL) in PBS, followed by gentle shaking for 4 min. In the absence of terpolymer, a qualitatively similar interfacial, anchored multiphase morphology was observed, indicating that stomatocytes play a decisive role in inducing this structure. However, these coacervates were generally unstable without terpolymer stabilization, as evidenced by frequent droplet collapse under bright-field microscopy (Figure S5a). Thus, while stomatocytes are essential for inducing interfacial, anchored multiphase organization, the terpolymer confers structural stability to the coacervate droplets (Figure S5).

In stomatocyte-only stabilized systems, the subdroplets exhibited pronounced enrichment in BSA and Q-Am, whereas Cm-Am remained homogeneously distributed (Figures 3a and S6). These observations indicate that subdroplet formation arises from stomatocyte-derived interfacial heterogeneities, as a homogeneous terpolymer membrane alone does not support interface-tethered multiphase separation. Incorporation of stomatocytes introduces discrete, localized electrostatic domains that perturb the otherwise uniform interfacial charge distribution, thereby directing the nucleation and stabilization of membrane-associated multiphase structures. Consistent with this interpretation, additional electrostatic interactions between negatively charged stomatocytes and positively charged Q–Am are evidenced by the marked enrichment of Q-Am within the subdroplets (Figure S7). Moreover, terpolymer incorporation yields significantly enhanced structural stability compared to systems lacking terpolymer.

**Figure 3:**
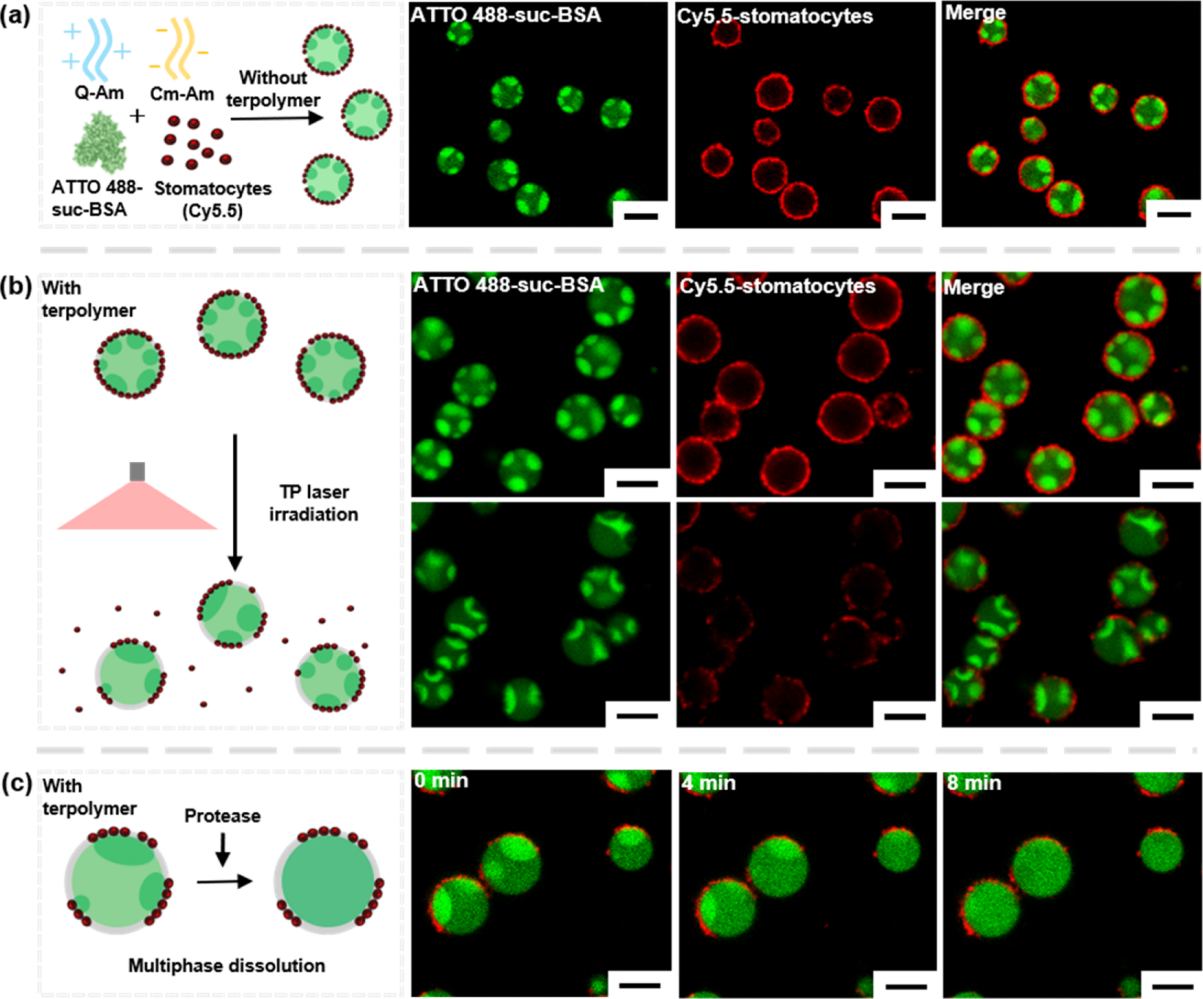
(a) CLSM images of multiphase-separated, stomatocyte-stabilized coacervates. Composition is 33 μL 1 mg/mL Cm-Am solution, 20 μL 4.76 mg/mL stomatocyte solution, 5 μL 3.33 mg/mL suc-BSA solution, and 66 μL 1 mg/mL Q-Am solution, which are mixed by shaking for 4 min. (b) CLSM images of multiphase-separated 0.40-Coas before two-photon (TP) laser irradiation (top) and after 1 min TP laser irradiation (bottom). The TP laser intensity is 0.3%. The gray dashed box indicates the region used to highlight the distribution of stomatocytes at the interface. Composition of 0.40-Coas: negatively charged amylose (Cm-Am; 33 μL, 1 mg/mL), positively charged amylose (Q-Am; 66 μL, 1 mg/mL), suc-BSA (5 μL, 3.33 mg/mL), stomatocytes (10 μL, 4.76 mg/mL) and terpolymer (5 μL, 25 mg/mL in PEG 350) in phosphate-buffered saline (PBS). (c) CLSM images of multiphase-separated 0.21-Coas at different time points after protease addition. Protease was added at 0 min, resulting in gradual disappearance of BSA-enriched subdroplets via BSA hydrolysis. Composition of 0.21-Coas: negatively charged amylose (Cm-Am; 33 μL, 1 mg/mL), positively charged amylose (Q-Am; 66 μL, 1 mg/mL), suc-BSA (5 μL, 3.33 mg/mL), stomatocytes (5 μL, 4.76 mg/mL) and terpolymer (5 μL, 25 mg/mL in PEG 350) in phosphate-buffered saline (PBS). In each panel, left: schematic illustration of the system; right: corresponding CLSM images. Red: Cy5.5-labeled stomatocytes; green: ATTO-488–conjugated suc-BSA. Scale bars: 5 μm.

Next, we systematically studied the distribution of each component within this multiphase system (Figure S7). The favorable electrostatic interaction between the positively charged Q-Am and negatively charged Cm-Am and suc-BSA induced multiphase separation, similar to previously described systems, where multiphase condensates are formed in a dilute phase.^41,42^ For Coas (without stomatocytes), Q-Am was strongly enriched in the same regions as the BSA-enriched subdroplets, whereas Cm-Am showed only weak enrichment in these subdroplets. This indicates that, compared to Cm-Am, the electrostatic interaction between Q-Am and BSA is stronger. Similar component distributions were observed in the 0.04-Coas, indicating that the introduction of a small number of stomatocytes had little impact on the overall multiphase system. As the density of stomatocytes at the interface of coacervates increased, more BSA-enriched subdroplets occurred in the system of 0.21-Coas and 0.40-Coas. Q-Am remained strongly enriched within the subdroplets, which were also enriched in BSA, whereas Cm-Am was distributed uniformly. This can be attributed to the electrostatic repulsion caused by the negatively charged stomatocytes at the interface, which exhibit nearly complete coverage of the coacervate interface (Figure S7a). Besides, quantitative analysis of fluorescence intensity of Cm-Am, Q-Am, and suc-BSA within subdroplets revealed that, as the interfacial stomatocytes’ density increased, the concentrations of BSA, Q-Am, and Cm-Am within subdroplets all decreased. This was due to the introduction of a large amount of highly negatively charged stomatocytes at the initial stage of LLPS, which altered the concentrations of each component involved in the multiphase separation. These results indicated that the density of stomatocytes at the coacervate interface affected the position, number, size, and composition of the subdroplets (Figure S7b).

### Dynamic Interaction between Stomatocytes and Subdroplets

In order to understand the dynamic interplay between stomatocytes and subdroplets, we manipulated either the stomatocytes or the subdroplets and observed the resulting changes in their spatial distribution. Upon two-photon (TP) irradiation (λ_ex_ = 800 nm) of the 0.40-Coas sample for ∼1 min, low-intensity TP laser (0.1%) illumination produced no observable changes in the droplets. In contrast, high-intensity (higher than 0.2%) TP irradiation led to a pronounced decrease in the number of stomatocytes at the coacervate interface, indicating their escape from the surface (Figure S8). This behavior arises from plasmonic heating generated by photoactivated AuNPs on the stomatocytes, which endows them with motile features and weakens their adsorption at the interface.^51^ As interfacial stomatocytes dissociated, their coverage transitioned from near-complete to patch-like. Concomitantly, the internal multiphase organization was altered: loss of interfacial stomatocytes reduced the electrostatic support for BSA-enriched subdroplets, promoting their fusion (Figure 3b). The departure of stomatocytes from the interface resulted in a marked decrease in the number of BSA-enriched subdroplets within the coacervates (Figure S9). The larger-sized BSA-enriched droplets formed after fusion tended to relocate at sites on the interface where there were still patches of stomatocytes (Figure 3b). The dynamic response of the system under localized, nonequilibrium perturbation induced by TP laser irradiation indicate that interfacial stomatocytes play a critical role in establishing and maintaining the BSA-enriched subdroplets within the coacervates, and that their displacement induces dynamic rearrangements of the internal multiphase architecture.

Conversely, we examined whether dynamic changes in the internal BSA-enriched droplets influenced the interfacial arrangement of the stomatocytes. Upon introducing an appropriate amount of protease into the multiphase system to degrade BSA, the BSA-enriched subdroplets disappeared. CLSM imaging revealed that the interfacial stomatocytes did not undergo any noticeable rearrangement despite the loss of the internal subdroplets (Figure 3c). Hence, alterations in the internal multiphase organization did not perturb the spatial positioning of stomatocytes at the interface, further confirming that the particles remained robustly anchored at the coacervate interface.^52^

In our system, multiphase separation is driven by electrostatic association between oppositely charged components. When we decreased the volumetric proportion of positively charged Q-Am (Cm-Am: Q-Am from 1:4 to 1:1), we observed the formation of more small-sized droplets within the coacervates (Figure S10). This behavior likely arises from the charge balance between the oppositely charged species, which limits the growth of nucleated droplets and stabilizes many small droplets. At very low Q-Am content (Cm-Am: Q-Am = 1:0.7), fewer but larger BSA-enriched droplets formed, and the coacervates exhibited morphological deformation. Under this condition, the pronounced net charge imbalance destabilizes subdroplets, promoting coalescence into larger subdroplets and generating uneven interfacial tension, which drives coacervate deformation. These observations are consistent with prior theoretical and experimental studies indicating that charge imbalance can modulate coacervate phase behavior, influence droplet size distributions, and slow coarsening dynamics in polyelectrolyte complex coacervates.^54,55^ Besides, when the succinylation degree of BSA was diminished, the resulting lower charge density was also not enough to support the growth of internal subdroplets after nucleation, and only uniform BSA distribution was observed (Figure S11).

### Stability of Interface-Anchored Controlled Multiphase Separation

Four coacervate systems with different degrees of stomatocyte coverage, i.e., Coas, 0.04-Coas, 0.21-Coas, and 0.40-Coas were stored at 4 °C. After 6 days, the BSA-enriched subdroplets within the Coas, 0.04-Coas, and 0.21-Coas systems gradually dissipated, whereas those in the 0.40-Coas samples remained stable and retained well-defined boundaries (Figure 4a). Quantification of the subdroplet number in freshly prepared 0.40-Coas and in samples aged for 6 days revealed no discernible difference (Figure 4b). The superior storage stability of the 0.40-Coas system suggests that near-complete coverage of the coacervate surface by the stomatocytes is essential for preserving the internal multiphase architecture, as the stomatocyte-covered surface layer provides sufficient electrostatic support to stabilize the subcompartmentalized structure.

**Figure 4:**
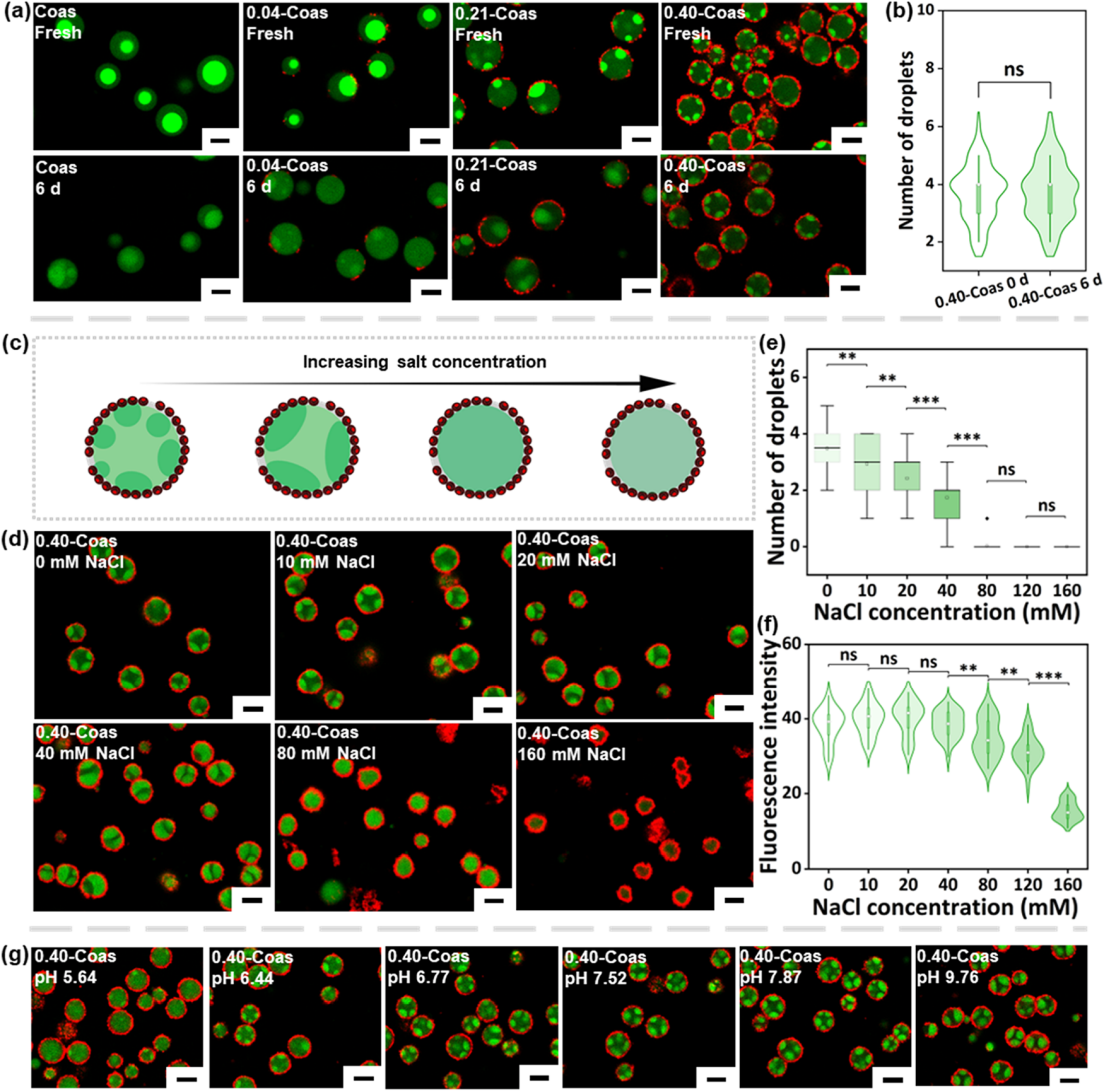
(a) CLSM images of fresh Coas, 0.04-Coas, 0.21-Coas, and 0.40-Coas, and the corresponding samples after 6 days. (b) Quantification of the number of BSA-enriched subdroplets in fresh 0.40-Coas and after 6 days. 50 coacervates were analyzed for each condition. (c) Schematic illustration of the evolution of the multiphase structure within 0.40-Coas upon increasing NaCl concentration. (d) CLSM images showing NaCl-induced changes in the multiphase structure of 0.40-Coas. (e) Quantification of the number of BSA-enriched subdroplets in 0.40-Coas incubated at different NaCl concentrations. 50 coacervates were analyzed for each condition. The box represents the interquartile range (IQR) between the first quartile (25%) and the third quartile (75%), with the horizontal line indicating the median. The whiskers extend to the minimum and maximum values within 1.5 × IQR of the quartiles. The mean value is indicated by a hollow square. Data points beyond this range are shown as outliers. (f) Fluorescence intensity of BSA-enriched droplets in 0.40-Coas as a function of NaCl concentration. Fluorescence intensity in BSA-loaded 0.40-Coas was measured in a ROI of identical area. For coacervates containing BSA-enriched subdroplets, the ROI was positioned within the subdroplets; for coacervates without subdroplets (uniform BSA distribution), the ROI was placed within the coacervates. Thirty coacervates were analyzed for each condition. (g) CLSM images showing pH-induced changes in the multiphase structure of 0.40-Coas. Red: Cy5.5-labeled stomatocytes; green: ATTO-488 conjugated suc-BSA. Scale bars: 5 μm. Images in panel (a) were processed using identical brightness and contrast settings to ensure comparability within the panel. Images in panels (d) and (g) were each processed using separate, internally consistent brightness and contrast settings. All images, except those used for fluorescence quantification in panel (f), were shown for visualization purposes only. Quantitative fluorescence analysis in panel (f) was performed using raw, unprocessed images from panel (d).

Having established the storage stability of the 0.40-Coas system, we next examined how varying ionic strengths influence the integrity of this multiphase system. The multiphase coacervates were first assembled under neutral conditions (pH 7.4), after which the ionic strength was adjusted *in situ* by varying the NaCl concentration in the system (Figure 4c). Therefore, the ionic strength-dependent evolution of BSA-enriched droplets reflects postassembly reorganization rather than de novo phase separation. As the NaCl concentration increased, the BSA-enriched subdroplets within 0.40-Coas began to undergo fusion. Quantification of the subdroplet number revealed that fusion was initiated at NaCl concentrations of 10 mM, leading to a modest reduction in droplet count; however, the fluorescence intensity of the BSA-enriched subdroplets remained largely unchanged until 40 mM NaCl, indicating that BSA enrichment was still maintained at this stage. At 80 mM NaCl, extensive fusion resulted in complete loss of subdroplet structure, yielding a homogeneous distribution of BSA throughout the coacervates (Figure 4d,e). The corresponding fluorescence intensity was markedly reduced compared to that in the BSA-enriched droplets, consistent with the loss of localized enrichment (Figure 4f). When the salt concentration was increased beyond 120 mM, BSA remained homogeneously distributed within 0.40-Coas, while its fluorescence intensity gradually decreased (Figure 4d–f). As the ionic strength is increased, the interactions between the scaffold polymers weaken, and as such the systems adjusts and the concentration is decreased.^34^ In comparison, the other samples (0.04-Coas and 0.21-Coas) displayed markedly lower tolerance to elevated ionic strength. Consistent with their shorter storage lifetimes, this behavior may originate from insufficient negative charge density at the coacervate interface, which is required to stabilize the BSA-enriched multiphase organization (Figure S12).

Following these observations of enhanced salt tolerance, the effect of pH was investigated. Multiphase coacervates were first assembled under neutral conditions, after which the pH was adjusted *in situ*. 0.40-Coas also exhibited a markedly broader pH stability window (pH 6.44–9.76) (Figure 4g). In contrast, for 0.04-Coas and 0.21-Coas, pronounced fusion of BSA-enriched droplets occurred as the pH approached 8, leading to the disruption of the multiphase organization and a homogeneous distribution of BSA within the coacervates (Figure S13). Notably, under acidic conditions (pH 5.64), BSA-enriched droplets formed in 0.40-Coas became significantly smaller compared to those observed at neutral pH, in stark contrast to the droplet coalescence observed under alkaline conditions (pH 9.76) (Figure 4g). Lowering the pH induced similar size changes for 0.04-Coas and 0.21-Coas (Figure S13). Upon decreasing the pH to 5.64, the reduced net negative charge of suc-BSA weakens its electrostatic association with positively charged Q-amylose, leading to partial droplet shrinkage and fragmentation into smaller domains. In contrast, increasing the pH to 9.76 strengthens electrostatic attraction, promoting droplet coalescence and the formation of larger BSA-enriched domains.

We next examined the temperature stability of 0.40-Coas. A temperature-controlled heating stage integrated with a confocal microscope and a temperature sensor was used to enable homogeneous and continuous temperature regulation of the entire sample, together with real-time monitoring. The 0.40-Coas multiphase system exhibited remarkable stability, maintaining its phase-separated structure when subjected to rapid heating from 20 to 40 °C and subsequent cooling back to 20 °C at a consistent rate of 30 °C/min (Figure 5a). This structural integrity was preserved even after three consecutive heating–cooling cycles (Figures 5a and S14). Furthermore, the stomatocyte-supported multiphase separation system demonstrated sustained stability for over 10 min at 40 °C (Figures 5a and S15).

**Figure 5:**
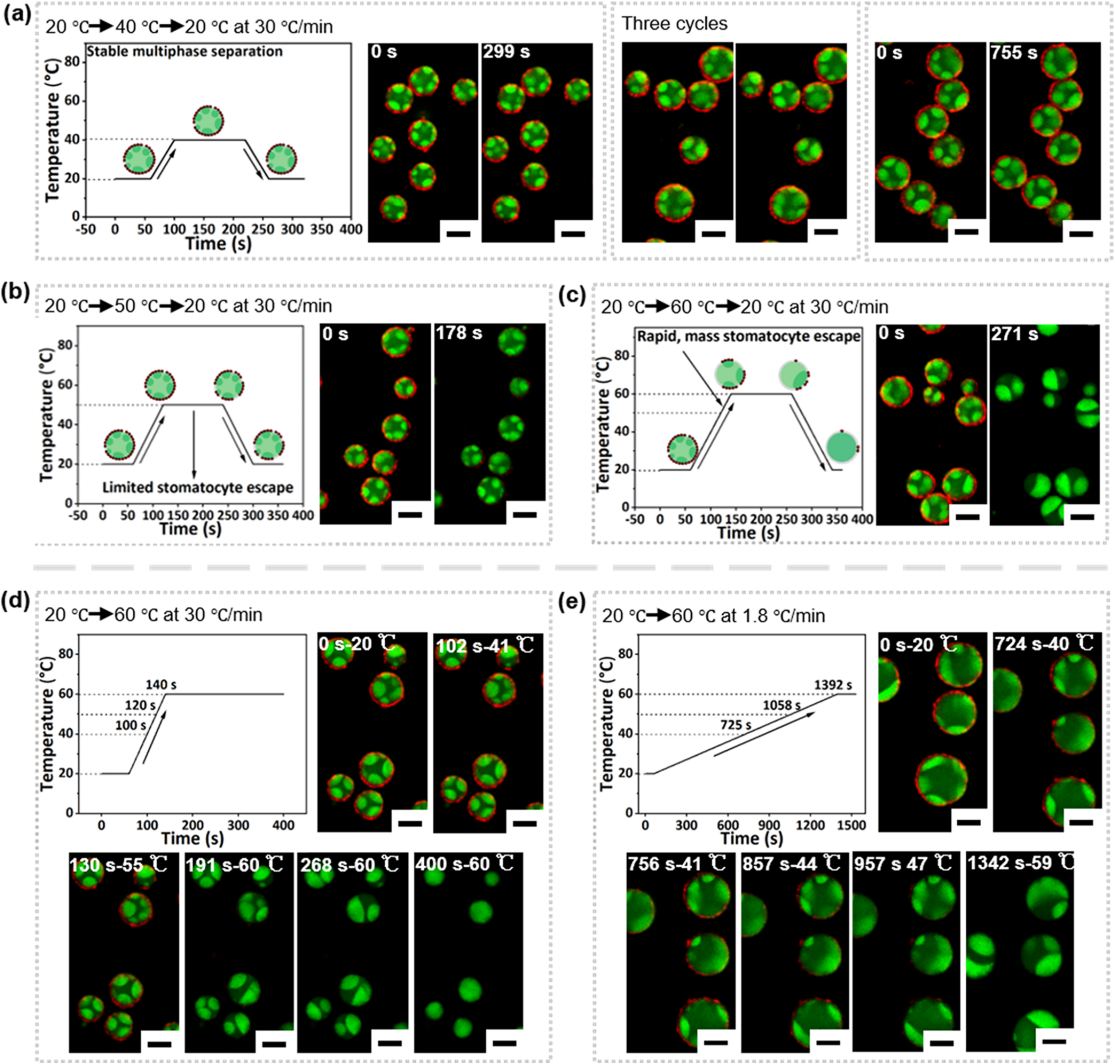
CLSM images showing temperature-induced changes in the multiphase structure of 0.40-Coas. (a) Temperature response at 40 °C: (left) heating from 20 to 40 °C (2 min hold) followed by cooling to 20 °C; (middle) after three heating–cooling cycles; (right) heating from 20 to 40 °C with a hold time >10 min. Heating and cooling rates were 30 °C/min. (b, c) Temperature response at elevated temperatures: (b) heating to 50 °C (2 min hold) then cooling to 20 °C; (c) heating to 60 °C (2 min hold) then cooling to 20 °C; heating and cooling rates were 30 °C/min. (d, e) Effect of heating rate to 60 °C: (d) 30 °C/min; (e) 1.8 °C/min; samples held at 60 °C after heating. Red: Cy5.5-labeled stomatocytes; green: ATTO-488 conjugated suc-BSA. Scale bars: 5 μm. All images were processed with identical brightness and contrast settings and were intended solely for visualization; they were not used for quantitative fluorescence analysis.

However, when the system temperature was increased from 20 to 50 °C and subsequently cooled back to 20 °C at the same rate of 30 °C/min, we observed that, although the overall multiphase-separated structure remained intact, the stomatocytes initially localized at the interface gradually escaped from the terpolymer membrane at 50 °C. This behavior was evidenced by a pronounced decrease in red fluorescence intensity at the interface (Figures 5b and S16). Elevated temperature is expected to weaken electrostatic interactions responsible for stomatocyte interfacial adsorption and diminish their trapping within the terpolymer membrane, thereby promoting the release of stomatocytes from the coacervate interface.^52^ Consistent with the localized heating results in Figure 3b, increased thermal energy promotes the detachment of interfacial stomatocytes, thereby reducing electrostatic stabilization. While plasmonic heating induces rapid and localized disruption, global heating leads to a more gradual and uniform response. Notably, the cooling process did not reverse this change, indicating that stomatocyte escape was irreversible under these conditions. Although some stomatocytes escaped, those remaining at the interface were sufficient to temporarily sustain the multiphase structure (Figures 5b and S17). After approximately 9 min of incubation at 50 °C, the stomatocytes had completely escaped, leading to gradual fusion among BSA-enriched droplets within the multiphase system (Figure S17).

When the system temperature was further increased from 20 to 60 °C at a rate of 30 °C/min, we observed that the majority of stomatocytes rapidly escaped from the interface. This process was accompanied by a pronounced decline in red fluorescence intensity and the initiation of fusion among multiple BSA-enriched droplets within the coacervates (Figures 5c, S18, and S19). Unlike temperature elevation to 50 °C, complete escape of stomatocytes at temperatures exceeding 50 °C prevented the maintenance of the internal multiphase structure. Notably, despite the extensive escape of stomatocytes from the terpolymer membrane, the residual fluid terpolymer membrane remained sufficient to maintain the overall structural integrity of the coacervates. Following the complete escape of the stomatocytes, the coacervate surface became exclusively covered by the terpolymer and exhibited a noticeable reduction in size (Figure S18b). Stomatocyte escape increases the effective surface tension and removes steric or active stabilization at the interface, thermodynamically driving interfacial area minimization and coacervate compaction while preserving overall coacervate integrity. Concurrent release of internal components and reorganization of the terpolymer layer further promote this shrinkage.

We further examined the influence of heating and cooling rates on stomatocyte release and multiphase structure. At a rapid heating rate of 30 °C/min, most stomatocytes remained stably anchored at the interface before the temperature reached 50 °C (120 s) (Figures 5d and S19). A similar behavior was observed at 12 °C/min, where at 210 s (50 °C) most stomatocytes had not yet escaped (Figure S20). In contrast, when the heating rate was reduced to 1.8 °C/min, the majority of stomatocytes had already detached before the temperature reached 50 °C (Figures 5e and S21). This behavior is consistent with thermally activated kinetics and a time–temperature dose effect: detachment is governed by processes with finite activation barriers, and slower heating provides sufficient cumulative “thermal dose” for these processes to proceed at lower apparent temperatures.^56,57^

### Mechanistic Model for Stomatocytes-Regulated Subcompartment Formation

Based on the above results, we propose a mechanistic model for stomatocyte-regulated subcompartment formation within terpolymer-stabilized amylose-based coacervates. In this system, multiphase separation is primarily driven by electrostatic association between oppositely charged components, particularly the strong interaction between positively charged Q-Am and negatively charged suc-BSA, leading to the formation of BSA-enriched subdroplets within the coacervate interior.

The incorporation of negatively charged stomatocytes at the coacervate interface introduces discrete, localized electrostatic patches that break the otherwise homogeneous interfacial landscape. These stomatocyte-rich regions preferentially attract Q-Am, thereby locally enhancing electrostatic association with suc-BSA and nucleating BSA-enriched subdroplets at the inner interface. As a result, subdroplet formation becomes spatially correlated with the distribution of stomatocytes decorating the coacervate surface, as evidenced by the positional correspondence observed in both 2D and 3D fluorescence imaging.

Increasing the density of interfacial stomatocytes amplifies this effect by generating a larger number of electrostatically favorable nucleation sites, leading to a higher number of smaller BSA-enriched subdroplets. Conversely, sparse or patchy stomatocyte coverage provides insufficient electrostatic support, resulting in fewer and less stable subdroplets. These observations demonstrate that the density and spatial organization of interfacial stomatocytes dictate the number, size, and positioning of internal subcompartments. Importantly, the stomatocyte-guided formation of interfacial subdroplets is observed in both terpolymer-containing and terpolymer-free systems, confirming that stomatocytes are the primary determinant of nucleation and spatial organization. However, the presence of the terpolymer significantly enhances the mechanical stability of the coacervate interface, thereby improving the robustness of the overall multiphase architecture.

Dynamic perturbation experiments further support the central role of stomatocytes in stabilizing the multiphase architecture. Photoinduced dissociation of stomatocytes from the interface reduces local electrostatic stabilization, triggering fusion of BSA-enriched subdroplets, whereas enzymatic degradation of BSA eliminates subdroplets without affecting stomatocyte positioning. This asymmetry confirms that interfacial stomatocytes act as the primary structural regulators of subcompartment formation, while the internal droplets remain dynamically responsive to changes in electrostatic interactions.

Electrostatic balance within the coacervate further modulates subdroplet morphology and stability. Variations in the relative abundance of Q-Am and suc-BSA alter charge neutralization, influencing nucleation density, droplet growth, and coalescence behavior. Pronounced imbalance promotes fusion and interfacial deformation, consistent with established coacervate theory.

Finally, the near-complete coverage of the coacervate interface by stomatocytes in the 0.40-Coas system provides robust electrostatic support that leads to long-term stability and broadens tolerance to ionic strength and pH variations. Increasing the salt concentration or applying an extreme pH condition screens electrostatic interactions, leading to subdroplet fusion and eventual loss of multiphase organization. Together, these results establish a mechanistic framework in which interfacial electrostatic patterning by charged nanoparticles templates, stabilizes, and dynamically regulates membrane-associated multiphase separation within synthetic coacervates.

These principles of interface-templated multiphase separation, where local interfacial heterogeneity dictates the formation and stability of subcompartments, share conceptual similarities with natural membrane-associated condensates, such as synapsin-1 condensates that capture negatively charged lipid vesicles via electrostatic interactions.^58^

To evaluate the generality of the proposed interfacial templating mechanism, AuNPs-decorated polymersomes were employed as an alternative coating system in place of stomatocytes. As shown in Figure S22a, polymersome-coated interfaces similarly induced the formation of BSA-enriched subcompartments within the coacervate phase, indicating that the observed multiphase organization is not specific to stomatocyte morphology. Quantitative comparison of particle properties (Figure S22b) shows that polymersomes are larger and more negatively charged than stomatocytes, which likely affects their interfacial packing and electrostatic interactions with the coacervate matrix. While these particles remain capable of templating subdroplet nucleation, increased polymersome loading results in partial coacervate deformation and aggregation, suggesting a less favorable balance between interfacial adsorption and colloidal stability. Although AuNP-decorated stomatocytes exhibited superior performance under our experimental conditions, particularly in combination with the terpolymer for patchy membrane formation, they are not uniquely required for templated subcompartment formation. We therefore suggest that patchy interfacial organization represents a general mechanism, governed primarily by interfacial electrostatics and particle–coacervate interactions rather than specific nanoparticle geometry.

### Cargo Uptake of Interface-Anchored Controlled Multiphase Separation

As an example of how complex multiphase separation architectures can be used, we consider the case in which coacervates are used for effective cargo uptake. A series of coacervates (Coas, 0.04-Coas, 0.21-Coas, and 0.40-Coas) were prepared. Suc-BSA was not dye-labeled; instead, stomatocytes loaded with Cy5.5 were employed to distinguish among the different systems. First, we set out to load our cargo, green fluorescent protein (GFP, negatively charged and neutral). GFP was added to different multiphase coacervate samples (Coas, 0.04-Coas, 0.21-Coas, and 0.40-Coas). After 13 min, we tested the ability of these coacervates to take up neutral GFP and supercharged GFP with a net negative charge of 30 (−30GFP). Neutral GFP was excluded from all coacervate samples, with a higher exclusion effect at higher stomatocyte densities (Figures 6a and S23). We found that −30GFP was taken up by coacervates and also showed different compartmentalization enrichment effects in the different systems (Figure 6b). The fluorescence intensity of −30GFP in subcompartments for 5 representative coacervates was plotted over time (Figure 6c). We found that 0.21-Coas and 0.40-Coas sequestered the cargo into internal subcompartments substantially more quickly. After 3 min of adding −30GFP to the 0.21-Coas and 0.40-Coas samples, the uptake of −30GFP reached saturation. For Coas and 0.04-Coas samples, the uptake kinetics of −30GFP was slower. These results illustrate that coacervates with a larger interfacial area (0.21-Coas and 0.40-Coas) are faster in cargo uptake into subcompartments. On the other hand we also observed that the final fluorescence intensity of −30GFP in subcompartments of 0.21-Coas and 0.40-Coas was lower than that in Coas and 0.04-Coas, which is determined by the concentration of Q-Am within the different subcompartments. Higher concentrations of Q-Am within subcompartments of Coas and 0.04-Coas induce stronger −30GFP uptake ability (Figure S7). Thus, being able to control the coacervate architecture yields control over uptake ability and rate.

**Figure 6:**
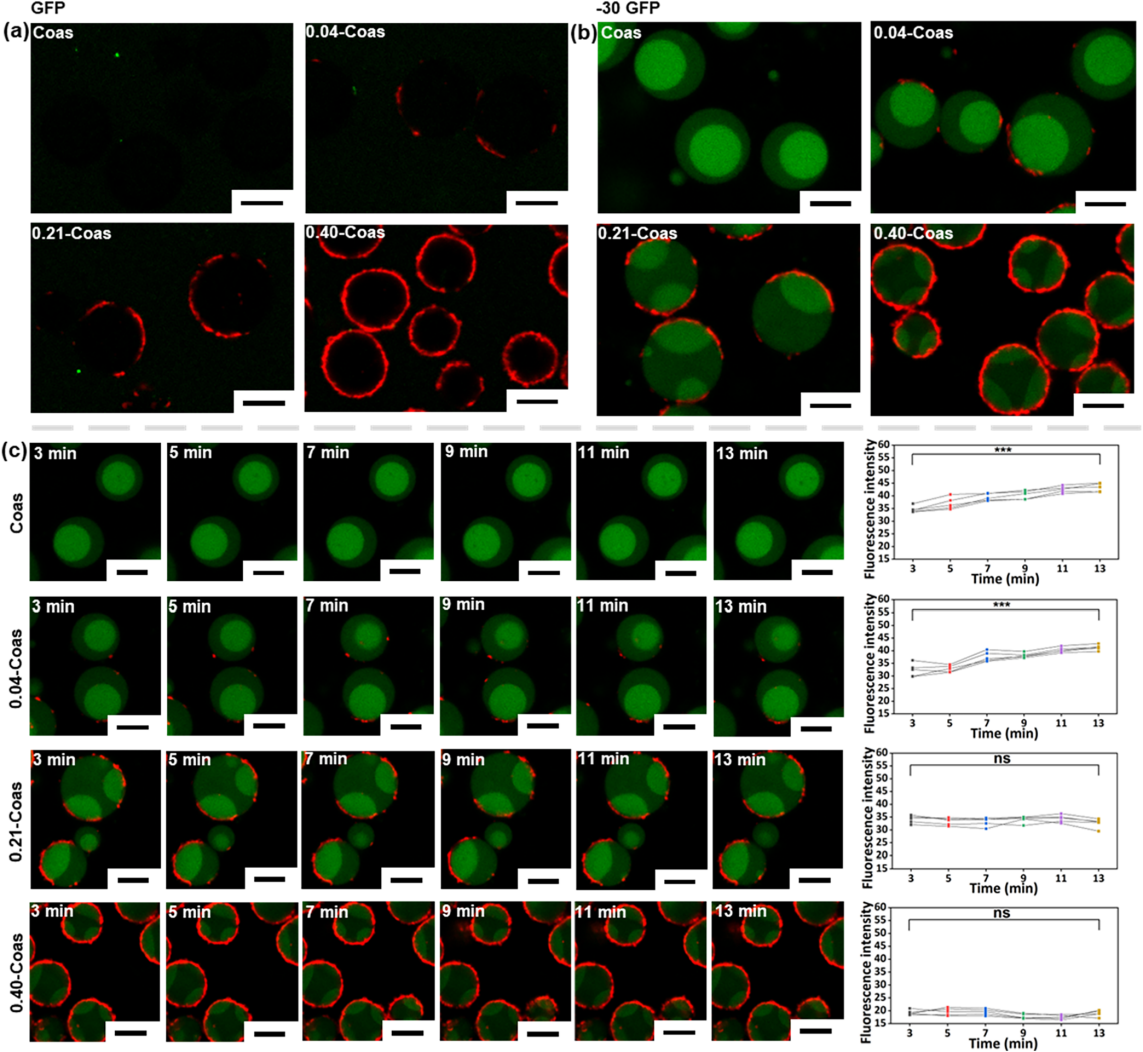
Control of cargo uptake by modulation of coacervate multiphase structure. CLSM images showing the uptake of GFP (a) and −30GFP (b) into Coas, 0.04-Coas, 0.21-Coas, and 0.40-Coas after 13 min of GFP or −30GFP incubation. (c) Time-lapse analysis of −30GFP fluorescence intensity in subcompartments of Coas, 0.04-Coas, 0.21-Coas, and 0.40-Coas, with quantification at 3, 5, 7, 9, 11, and 13 min. Five well-focused coacervate droplets of similar size were selected for each condition. For GFP and −30GFP uptake, 2 μL of 20 μM GFP solution was added to 100 μL coacervates and mixed. Red: Cy5.5-labeled stomatocytes; Green: GFP or −30GFP. Scale bars: 5 μm. All images were processed using identical brightness and contrast settings. Fluorescence quantification was performed using raw, unprocessed images from panel (c). Statistical significance between conditions is indicated by *** (*p* < 0.001), while ns indicates no significant difference.

### Programmable Chemical Communication between Protocell Populations

To investigate whether the programmable internal architecture of the coacervate protocells could regulate chemical communication, an enzymatic cascade reaction was established between two spatially separated protocell populations (Figure 7a). Glucose oxidase (GOX) was encapsulated within amylose-based coacervates, whereas Cy5-labeled horseradish peroxidase (HRP) was incorporated into programmable multiphase coacervates containing stomatocyte-directed BSA-enriched droplets (Figures S24 and S25). Then mixing two coacervates populations. Upon addition of glucose and *o*-phenylenediamine (*o*-PD), GOX generated hydrogen peroxide (H_2_O_2_), which diffused between protocell compartments and served as a chemical messenger to activate HRP-catalyzed oxidation of *o*-PD into fluorescent diaminophenazine (DAP). The progressive increase in DAP fluorescence confirmed efficient signal transfer between distinct protocell populations, demonstrating a protocell communication pathway mediated by diffusible molecular intermediates (Figure 7).

**Figure 7:**
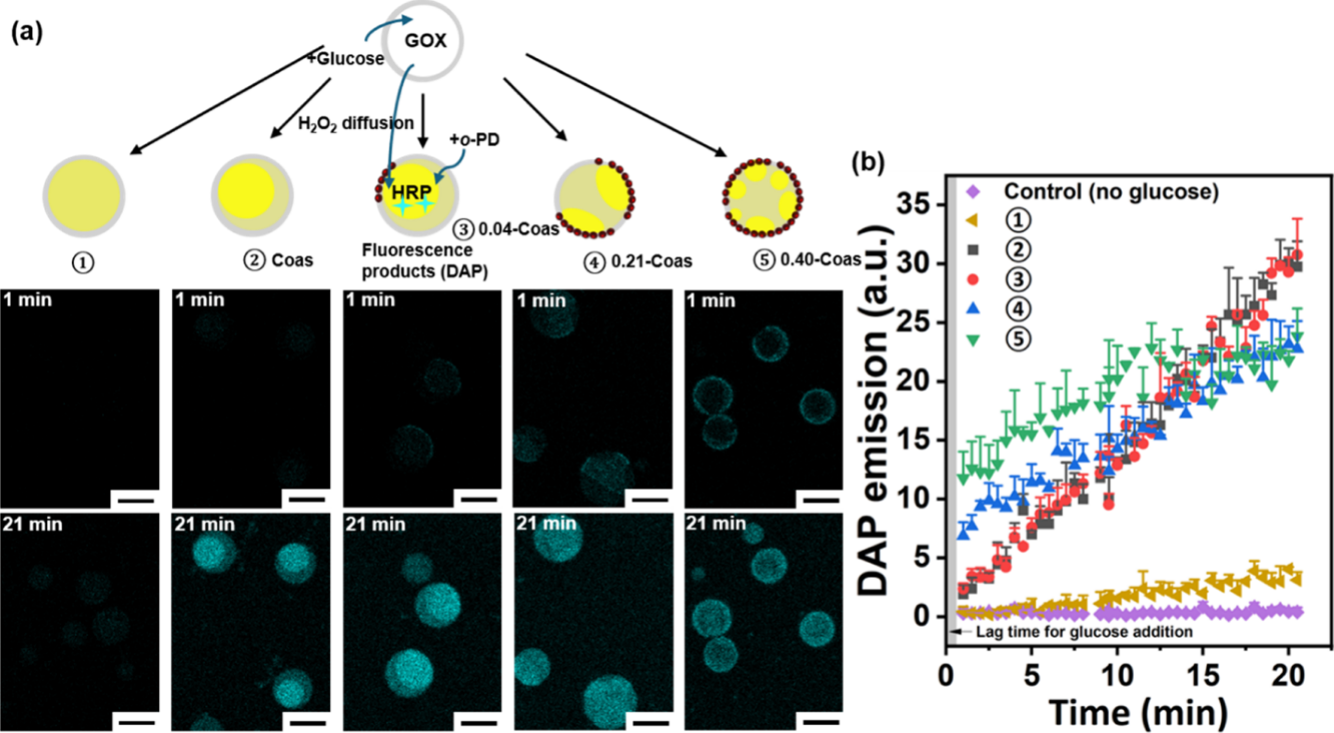
Stomatocyte-guided spatial organization regulates chemical communication between protocell populations. (a) Schematic illustration of chemical communication between two protocell populations. Glucose oxidase (GOX) encapsulated within amylose-based coacervates catalyzes the oxidation of glucose, generating H_2_O_2_ as a diffusible signaling molecule. The generated H_2_O_2_ is subsequently transferred to neighboring horseradish peroxidase (HRP)-loaded coacervates, where HRP catalyzes the oxidation of *o*-phenylenediamine (*o*-PD) into fluorescent diaminophenazine (DAP). Representative confocal fluorescence images showing DAP formation in homogeneous coacervates and programmable multiphase coacervates with distinct stomatocyte-guided internal organizations (Coas, 0.04-Coas, 0.21-Coas, and 0.40-Coas) at 1 and 21 min after glucose addition. Scale bars: 5 μm. (b) Time-dependent evolution of DAP fluorescence intensity measured in the HRP-enriched regions of different HRP-loaded coacervate populations following glucose addition. The control experiment performed in the absence of glucose exhibited negligible fluorescence increase, confirming that DAP formation resulted from the GOX/HRP enzymatic cascade (Mixing GOX-loaded coacervates and HRP-loaded 0.04-Coas). Data are presented as mean ± SD (*n* = 3).

We next examined how the spatial organization of HRP affected the communication dynamics. In contrast to homogeneous coacervates, HRP in the programmable multiphase coacervates was selectively concentrated within stomatocyte-templated BSA-rich subcompartments located at interfacial region inside the protocells (Figure S25). Notably, coacervates possessing interfacially localized HRP-rich droplets exhibited a substantially faster increase in DAP fluorescence during the early stages of the reaction (1 min; lag time) (Figure 7b), indicating accelerated activation of the cascade process. We attribute this behavior to the reduced diffusion distance between externally generated H_2_O_2_ and the membrane-associated catalytic domains, allowing the enzyme-rich droplets to function as localized reaction hotspots. Such spatial positioning therefore facilitates rapid interception of incoming chemical signals and promotes faster initiation of protocell communication.

Interestingly, the kinetic advantage associated with interfacial localization became less pronounced at longer reaction times. As H_2_O_2_ gradually diffused throughout the coacervate phase, the influence of subcompartment position diminished and the overall catalytic output became increasingly dependent on enzyme loading and distribution within the protocells (Figure 7b). Consequently, different coacervate populations displayed distinct temporal reaction profiles despite converging communication pathways. Furthermore, all multiphase coacervates outperformed homogeneous coacervates, consistent with the enhanced sequestration of HRP within the BSA-enriched droplets. The resulting increase in local enzyme concentration generated higher catalytic efficiencies and amplified signal transduction between protocell populations.

Together, these findings demonstrate that stomatocyte-guided subcompartmentalization not only enables programmable spatial localization of enzyme-containing droplets but also regulates the dynamics of chemical communication. By controlling where catalytic domains emerge within protocells, the timing and efficiency of interprotocellular signal transmission can be modulated, providing a life-like mechanism through which structure encodes function in synthetic protocell communities.

## Conclusion

In summary, this work demonstrates that interfacial electrostatic heterogeneity can direct multiphase separation in amylose-based coacervates, providing spatial control over subcompartment formation near membrane interfaces. By embedding negatively charged stomatocytes within a terpolymer membrane, we create localized charged domains that enable the formation of highly stable and tunable multiphase systems, resilient to environmental variations such as changes in ionic strength and pH. The membrane-anchored multiphase architecture also exhibits dynamic responsiveness to temperature and ionic strength. Furthermore, the expanded interfacial area of the engineered multiphase system markedly enhances cargo uptake, highlighting the practical utility of localized interfacial control in this specific system. Beyond structural organization, the programmable positioning of enzyme-containing subcompartments enables regulation of interprotocellular chemical communication, where spatially localized catalytic domains modulate the timing and efficiency of signal transduction. These findings establish interfacial electrostatic heterogeneity as a key organizing factor within this coacervate system, offering guidance for the rational design of membrane-associated multiphase architectures with tailored functionality and emergent communication behaviors.

## Supplementary Material



## Notes

Materials and instruments, synthesis and methods, supporting figures S1–S25, supporting references (PDF)
