# Supplementary material for: Patchy Membrane-Directed Multiphase Complex Coacervation
Source: J Am Chem Soc. 2026 Jul 27;148(31):33945–58. doi: 10.1021/jacs.6c11826 (PMC13474923; doi:10.1021/jacs.6c11826)
Supplement: Supplementary file 1 [file ja6c11826_si_001.pdf]

Supplementary information

Patchy Membrane-Directed Multiphase Complex Coacervation

Siwen Sun[†], Nadia Erkamp[†], Sander G. A. M. Huisman[†], Alexander B. Cook[†], Arjan Hazegh Nikroo[†], Zhuping Yin[†], Jianhong Wang[†], Madelief A. M. Verwiel[†], Jan C. M. van Hest[†,]*

[†]Bio-Organic Chemistry, Departments of Biomedical Engineering and Chemical Engineering & Chemistry, Institute for Complex Molecular Systems, Eindhoven University of Technology, 5600 MB Eindhoven, The Netherlands.

Contents

1. Materials

2. Instruments

3. Synthesis and methods

3.1 Synthesis of modified amyloses

3.2 Synthesis of terpolymer

3.3 Synthesis of $PEG_{22}PDLLA_{95}$ and $PEG_{44}PDLLA_{95}$

3.4 Preparation of PEG-PDLLA stomatocytes

3.5 Preparation of AuNPs-coated stomatocytes

3.6 Preparation of succinylated proteins

3.7 Preparation of multiphase coacervates

3.8 pH, salt, and temperature stability of multiphase coacervates

3.9 Protease-treated multiphase coacervates

3.10 Green Fluorescent Protein variants

3.11 GFP cargo uptake of multiphase coacervates

3.12 Preparation of enzyme-loaded coacervates and determination of enzyme encapsulation efficiency

3.13 Conditions for enzyme cascade assays

4. Supplementary figures

Figure S1. CLSM images of stomatocyte-stabilized amylose-based coacervates.

Figure S2. CLSM images of stomatocytes inserted into terpolymer-stabilized amylose-based coacervates.

Figure S3. Quantification of the number of BSA-enriched subdroplets within individual coacervates.

Figure S4. 2D and 3D z-stack micrographs of representative 0.04-Coas and 0.21-Coas droplets.

Figure S5. CLSM images of stomatocyte-stabilized multiphase coacervates and stomatocyte insert into terpolymer-stabilized multiphase coacervates.

Figure S6. CLSM figures of multiphase-separated stomatocyte-stabilized coacervates (in the absence of terpolymer).

Figure S7. CLSM figures of Coas, 0.04-Coas, 0.21-Coas, and 0.40-Coas. Quantification of fluorescence intensity of suc-BSA, Q-Am, and Cm-Am within Coas, 0.04-Coas, 0.21-Coas, and 0.40-Coas.

Figure S8. CLSM images of multiphase-separated 0.40-Coas before two-photon (TP) laser irradiation and after 1-min TP laser irradiation.

Figure S9. Quantification of the number of BSA-enriched subdroplets within 0.40-Coas before TP laser irradiation and after 1-min 0.3% TP laser irradiation.

Figure S10. CLSM images of multiphase-separated 0.40-Coas formed at different volume ratios of Cm-Am to Q-Am (1:5.7-1:0.25).

Figure S11. CLSM images of multiphase-separated 0.40-Coas formed using suc-BSA with different charge densities. Zeta potentials of suc-BSA prepared with different succinylation time.

Figure S12. CLSM images illustrating NaCl-induced evolution of the multiphase architecture in 0.04-Coas and 0.21-Coas.

Figure S13. CLSM images illustrating pH-induced evolution of the multiphase architecture in 0.04-Coas and 0.21-Coas.

Figure S14. CLSM images showing temperature-induced changes in the multiphase structure of 0.40-Coas after three heating–cooling cycles between 20 and 40 °C (heating and cooling rates: 30 °C /min).

Figure S15. CLSM images showing temperature-induced changes in the multiphase structure of 0.40-Coas upon heating from 20 to 40 °C at 30 °C /min with a hold time >10 min.

Figure S16. CLSM images showing temperature-induced changes in the multiphase structure of 0.40-Coas upon heating from 20 to 50 °C (2 min hold) followed by cooling to 20 °C. Heating and cooling rates were 30 °C /min.

Figure S17. CLSM images showing temperature-induced changes in the multiphase structure of 0.40-Coas upon heating from 20 to 50 °C at 30 °C /min with a hold time >10 min.

Figure S18. CLSM images showing temperature-induced changes in the multiphase structure of 0.40-Coas upon heating from 20 to 60 °C (2 min hold) followed by cooling to 20 °C. Heating and cooling rates were 30 °C /min. Size analysis of coacervates before stomatocyte escape and after stomatocyte escape.

Figure S19. CLSM images showing temperature-induced changes in the multiphase structure of 0.40-Coas upon heating from 20 to 60 °C at 30 °C /min with a hold time >10 min.

Figure S20. CLSM images showing temperature-induced changes in the multiphase structure of 0.40-Coas upon heating from 20 to 50 °C at 12 °C /min with a hold time >10 min, and upon heating from 20 to 60 °C at 12 °C /min with a hold time >10 min.

Figure S21. CLSM images showing temperature-induced changes in the multiphase structure of 0.40-Coas upon heating from 20 to 50 °C at 1.8 °C /min with a hold time >10 min, and upon heating from 20 to 60 °C at 1.8 °C /min with a hold time >10 min.

Figure S22. CLSM images of multiphase-separated polymersome-stabilized coacervates in the presence of terpolymer. Size and zeta potential of AuNPs decorated polymersomes and stomatocytes.

Figure S23. Uptake efficiency of Coas, 0.04-Coas, 0.21-Coas, and 0.40-Coas for neutral-charged GFP.

Figure S24. CLSM images of GOX-loaded terpolymer-stabilized amylose-based coacervates.

Figure S25. Stomatocyte-guided spatial organization regulates chemical communication between protocell populations.

5. References

1. Materials

All materials were ordered from Sigma-Aldrich without further purification unless otherwise stated.

For PEG-PDLLA synthesis:

Monomethoxy poly(ethylene glycol) 1 and 2 kDa were purchased from Rapp Polymere and freeze-dried before use. D,L-lactide (DLL) was purchased from Acros Organics. 1,4- Dioxane and tetrahydrofuran (THF) were obtained from Biosolve Chimie. Dialysis Membrane MWCO (12,000 - 14,000 Da) was supplied from Spectra/Pro®.

For the preparation of terpolymer:

Monomethoxy poly(ethylene glycol) 1 and 2 kDa were purchased from Rapp Polymere, trimethylene carbonate was purchased from TCI Europe.

Terpolymer was kindly provided by Sander G. A. M. Huisman. Additional details can be found in the Synthesis and Methods section

For the preparation of modified amylose derivatives:

Amylose (12-16 kDa) was supplied by Carbosynth and 3-chloro-2- hydroxypropyltrimethyl ammonium chloride (65 wt% in water) was supplied by TCI Europe.

DBCO-Cy5.5, sulfo-Cyanine3 NHS ester and sulfo-Cyanine5 NHS ester were purchased from Lumiprobe. Zeba™ Spin Desalting Columns (7K MWCO, 0.5 Ml) and phosphate-buffered saline (PBS) were procured from Thermo Fisher Scientific.

Two green fluorescent protein (GFP) variants were kindly provided by Arjan Hazegh Nikroo: a super folder GFP (GFP) and a supercharged GFP with net 30 negatively charged residues (-30GFP). Additional details can be found in the Synthesis and Methods section.

Ultrapure Milli-Q (Millipore) water (18.2 MΩ·cm) was used in this work.

2. Instruments

Confocal laser scanning microscopy (CLSM):

Confocal laser scanning microscopy (Leica TCS SP8) was used for visual analysis of coacervates. The system was equipped with a 488 nm laser (used for FITC), 552 nm laser (used for Nile red and Cy3), 638 nm laser (used for Cy5 and Cy5.5) and a hybrid detector (HyD). Cooling and heating experiments were performed with a TS102SI Instec rapid heating and cooling stage.

Two Photon-Confocal Laser Scanning Microscopy (TP-CLSM):

Two-photon laser activation was performed by using a CLSM (Leica TCS SP8X) equipped with 800 nm two-photon laser source (Chameleon Vision, Coherent, USA).

3. Synthesis and methods

3.1 Synthesis of modified amyloses

Both quaternized (Q-Am) and carboxymethylated (Cm-Am) amylose were prepared in accordance with previously published procedures[1, 2].

Q-Am was prepared by dissolving 1.5 g of amylose and 2.78 g of NaOH in 14.25 mL of Milli-Q water at 35 °C. After complete dissolution of the amylose, 11.64 mL of 3-chloro-2-hydroxypropyltrimethylammonium chloride solution (60 wt% in water) was added dropwise to the stirring reaction mixture. The reaction was allowed to proceed overnight under continuous stirring. Subsequently, the mixture was neutralized with acetic acid and precipitated into 200 mL of cold ethanol. The resulting precipitate was collected, re-dissolved in Milli-Q water, and extensively dialyzed against water using regenerated cellulose dialysis tubing (Spectrum Labs, USA) with a molecular weight cut-off (MWCO) of 3.5 kDa. The purified product was then lyophilized to obtain Q-Am. Approximately 5 g of Q-Am was obtained, corresponding to a yield of about 80%.

Cm-Am was synthesized by dissolving 1.5 g of amylose and 3.6 g of NaOH in 15 mL of Milli-Q water at 70 °C. After complete dissolution of amylose, 2.7 g of chloroacetic acid was added, and the reaction mixture was stirred for 2 h. The reaction was then neutralized with acetic acid and precipitated into 200 mL of cold ethanol. The precipitate was collected, re-dissolved in Milli-Q water, and dialyzed extensively against deionized water using regenerated cellulose dialysis tubing (MWCO 3.5 kDa, Spectrum Labs, USA). The purified product was obtained by lyophilization. Approximately 3 g of Cm-Am was recovered (ca. 90% yield).

3.2 Synthesis of terpolymer

Synthesis of poly(ethylene glycol)-*b*-poly(ε-caprolactone-*g*-trimethylene carbonate)-*b*-poly(glutamic acid) (PEG_{44}-*b*-P(CL_{53}-*g*-TMC_{54})-*b*-$PGlu_{11}$) terpolymer was synthesized as described previously[2, 3].

ε-Caprolactone and trimethylene carbonate were polymerized via ring-opening polymerization, initiated by poly(ethylene glycol) monomethyl ether and catalyzed by methanesulfonic acid. The polymer's terminal alcohol was then modified via Steglich esterification with Boc-L-Phe-OH, yielding a primary amine after TFA deprotection. The poly(L-glutamic acid) block was subsequently introduced through the ring-opening polymerization of N-carboxyanhydride γ-benzyl L-glutamate, followed by hydrogenolysis.

3.3 Synthesis of $PEG_{22}PDLLA_{95}$ and $PEG_{44}PDLLA_{95}$

The reaction was carried out using previously published procedures[4].

Briefly, 0.1 mmol poly(ethylene) glycol (PEG) was weighed into a flame-dried 50 mL round bottom flask. D,L-lactide (DLLA, 95 eq.) was added. The dry toluene (ca. 50 mL) was then added to the flask and the solvent evaporated in order to dry the contents before polymerization. The dried reagents were then redissolved in dry DCM (13 mL, [monomer] = 0.5M) and DBU was added (0.5 equiv. with respect to [initiator]; 0.1 mmol = 15 μL) under argon. The reaction was stored at RT for around 2 hours, until there was no evidence of the monomer from the ^{1}H-NMR spectra. After completion was confirmed by ^{1}H-NMR spectroscopy, the reaction mixture was diluted using DCM and washed twice with 1 M $KHSO_4$ and once with brine before drying with Na_2SO_4, filtering and evaporating most of the solvent. The concentrated copolymer solution (in DCM) was then precipitated into ice cold diethyl ether (100 mL) and the remaining

wax was partially dried under nitrogen before dissolving in dioxane and lyophilisation to yield a white powder.

3.4 Preparation of PEG-PDLLA stomatocytes

The stomatocytes were prepared following previously published procedures[4].

In a 20 mL vial, PEG_{22}-$PDLLA_{95}$ and PEG_{44}-$PDLLA_{95}$ block copolymers (1:1 w/w%, 10 mg) were weighed in and dissolved in 1 mL THF/dioxane (volume ratio=1:4). The vial was equipped with a stirring bar and sealed with a rubber septum. A needle was inserted to release pressure. The solution was stirred at 900 rpm for 30 min, followed by addition of 1 mL ultrapure water with a syringe pump at a rate of 1 mL/h. The solution was then dialyzed overnight against 75 mM NaCl at 4 °C with an ultrapure water change after 1 h to yield stomatocytes. For fluorescent stomatocytes: Cy5.5-DBCO (0.01 mg) was added to the organic solvent mixture before dissolving the copolymers.

3.5 Preparation of AuNPs-coated stomatocytes

The AuNPs-coated stomatocytes were prepared in accordance with previously published procedures[5].

In a 20 mL vial, poly(acrylic acid) (PAA, 0.8 mg) and $HAuCl_4 \cdot 4H_2O$ (1.2 µL, 1 mg mL^{-1}) were added to 1 mL PEG-PDLLA stomatocytes solution. The mixture was stirred at a rate of 500 rpm for 10 min at RT. Then, a solution of $NaBH_4$ (1 mL, 5 mM) was added dropwise and the reaction mixture was stirred for another 10 min at RT. The solution was centrifuged down, supernatant was removed and MilliQ water was added. This solution was washed three times. Finally, the precipitate was redispersed in 1 mL Milli-Q. Samples were stored in the 4 °C fridge for further use.

3.6 Preparation of succinylated proteins

Bovine serum albumin (BSA), glucose oxidase (GOX), or horseradish peroxidase (HRP) were labelled with Atto 488, Sulfo-Cy3, and Sulfo-Cy5, respectively, and subsequently succinylated. Briefly, 5 mg of protein was dissolved in 2.0 mL of 100 mM $NaHCO_3$ (2.5 mg/mL). Atto 488-NHS ester (20 µL, 10 mM in DMSO), Sulfo-Cy3-NHS ester (10 µL, 10 mM in DMSO), or Sulfo-Cy5-NHS ester (10 µL, 10 mM in DMSO) was added to the BSA, GOX, or HRP solution, respectively. The mixtures were incubated overnight at 4 °C (500 rpm). Succinic anhydride was then added to a final concentration of 50 mM, and the reactions were allowed to proceed for an additional 2 h under light protection. Excess reagents and free dye were removed by dialysis. The dialyzed solution was concentrated using a centrifugal ultrafiltration device (MWCO 3 kDa, 12,000 rpm, 40 min) and further purified with Zeba™ Spin Desalting Columns (7K MWCO). The purified suc-BSA solution was aliquoted and stored at -20 °C. The concentration of suc-BSA was determined gravimetrically by lyophilizing a known volume of the purified solution and weighing the resulting residue, yielding a final concentration of 3.33 mg/mL. Purified dye-labeled succinylated GOX and HRP were lyophilized and stored at -20 °C. Before use, the lyophilized enzymes were reconstituted in Milli-Q water to a concentration of 2 mg/mL.

3.7 Preparation of multiphase coacervates

For stomatocyte-stabilized multiphase coacervates, Q-Am and Cm-Am were dissolved separately in phosphate-buffered saline (PBS) at a concentration of 1 mg/mL. Coacervation was induced by adding 66 µL of Q-Am solution to a 1.5 mL Eppendorf tube containing 33 µL of Cm-Am solution, 5 µL 3.33 mg/mL suc-BSA solution and 20 µL 4.76 mg/mL stomatocyte solution while stirring in an Eppendorf tube shaker at 1500 rpm for 4 min. For preparation of

stomatocytes inserted into terpolymer-stabilized multiphase coacervates, coacervation was induced by mixing the solutions of 66 μL of Q-Am , 33 μL of Cm-Am, 5 μL 3.33 mg/mL suc-BSA solution and different volumes of stomatocyte solution in an Eppendorf tube shaker at 1500 rpm for 4 min. After 4 min of stirring, 5 μL of terpolymer stock solution (25 mg/mL in PEG350) was slowly added to stabilize the coacervate droplets.

3.8 pH, salt, and temperature stability of multiphase coacervates

Multiphase coacervates were prepared as described. For pH stability, the coacervate suspension (50 μL) was transferred to an Ibidi 18 well μ-slide. Then PBS buffer (50 μL) with different pH was added and measured with CLSM. The final pH was detected by a pH meter. For salt stability, 1 μL NaCl solution with different concentrations was added to 50 μL coacervate solution, the final NaCl concentration was 0, 10, 20, 40, 80, 120, and 160 mM, respectively. Cooling and heating experiments were performed with a TS102SIInstec rapid heating and cooling stage. 30 μL coacervate solution was added to the well for observation.

3.9 Protease-treated multiphase coacervates

Fresh multiphase-separated 0.21-Coas samples were prepared. To 50 μL of the 0.21-Coas solution, 2 μL of protease Type XIV from *Streptomyces griseus* (5 mg/mL; Sigma–Aldrich) was added. The changes in multiphase separation over time were then monitored using CLSM.

3.10 Green Fluorescent Protein variants

Two green fluorescent protein(GFP) variants were used; a super folder GFP (GFP) and a supercharged GFP with net 30 negatively charged residues (-30GFP). The proteins were expressed in *E. Coli* similar to previously described methods[6].

3.11 GFP cargo uptake of multiphase coacervates

Multiphase coacervates were prepared as described. The coacervate suspension (100 µL) was transferred to an Ibidi 18 well µ-slide. Then GFP or -30GFP (2 µL, 20 µM, PBS, pH 7.4) was added to the well and the uptake was measured with CLSM.

3.12 Preparation of enzyme-loaded coacervates and determination of enzyme encapsulation efficiency

For the preparation of HRP-loaded coacervates, Q-Am and Cm-Am were separately dissolved in phosphate-buffered saline (PBS) at a concentration of 1 mg/mL. Coacervation was induced by adding 66 µL of Q-Am solution to a 1.5 mL Eppendorf tube containing 33 µL of Cm-Am solution, 5 µL 3.33 mg/mL suc-BSA solution and 4.76 mg/mL stomatocyte solution (0 µL, 1 µL, 5 µL, and 10 µL) while stirring in an Eppendorf tube shaker at 1500 rpm for 4 min. Subsequently, terpolymer stock solution (5 µL, 25 mg/mL in PEG350) was added to stabilize the coacervate droplets. For the preparation of GOX-loaded coacervates, Q-Am solution (66 µL) was added to a 1.5 mL Eppendorf tube containing Cm-Am solution (33 µL) and Cy3-GOX solution (1 µL, 2 mg/mL) or unlabeled succinylated GOX at an equivalent concentration. The resulting mixture was agitated at 1500 rpm for 4 min, followed by the addition of terpolymer stock solution (5 µL, 25 mg/mL in PEG350) to stabilize the coacervate droplets.

To determine the enzyme encapsulation efficiency, Cy5-HRP-loaded and Cy3-GOX-loaded coacervate dispersions were allowed to stand undisturbed for 48 h to ensure complete sedimentation of the coacervate droplets. The supernatants were then carefully collected, and the fluorescence intensities of Cy5-HRP and Cy3-GOX were measured. The concentrations of unencapsulated enzymes remaining in the supernatants were determined using calibration curves correlating fluorescence intensity with enzyme concentration. The encapsulation

efficiencies were subsequently calculated from the difference between the initial amount of enzyme added and the amount detected in the supernatant. The encapsulation efficiency of GOX in homogeneous coacervates was 42 ± 1%, while that of HRP was 46 ± 4%. For compartmentalized HRP-loaded coacervates, encapsulation efficiencies of 82 ± 8%, 81 ± 3%, 62 ± 8%, and 38 ± 11% were obtained for Coas, 1-Coas, 5-Coas, and 10-Coas, respectively.

3.13 Conditions for enzyme cascade assays

Protocells were mixed at a 1:1 volume ratio (total volume: 60 μL) in an 18-well microscopy slide and allowed to settle for several minutes. An appropriate imaging region containing both GOX- and HRP-loaded protocells was selected for monitoring the reaction. Subsequently, 1 μL of 200 mM *o*-phenylenediamine (*o*-PD) was gently added to the well without disturbing the sample. To initiate the enzymatic cascade, 1 μL of 1 M glucose was then added, and time-lapse imaging was started immediately. Time-lapse fluorescence images were acquired at 30 s intervals. Image analysis was performed using ImageJ. For each condition, three representative protocells were selected. The mean fluorescence intensity of diaminophenazine (DAP), which produced in HRP-enriched regions, was quantified over time.

Supplementary figures

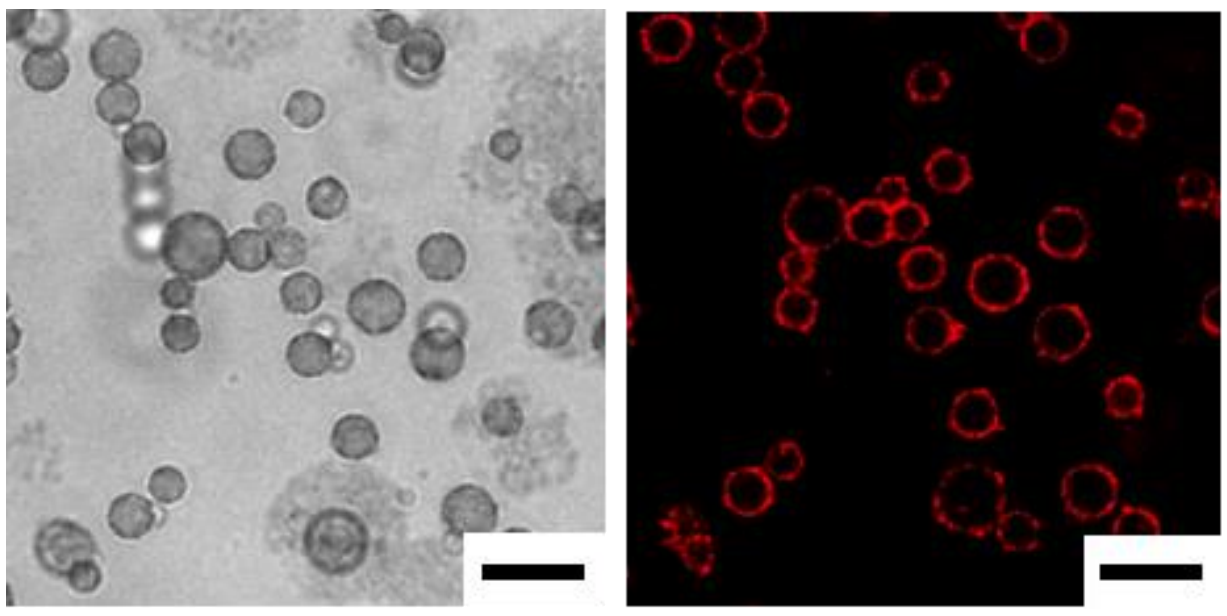

Figure S1. CLSM images of stomatocyte-stabilized amylose-based coacervates. Composition is 33 µL 1 mg/mL Cm-Am solution, 20 µL 4.76 mg/mL stomatocyte solution, and 66 µL 1 mg/mL Q-Am solution, which are mixed by shaking for 4 min. Stomatocytes are loaded with Cy5.5 (red). Scale bars represent 10 µm.

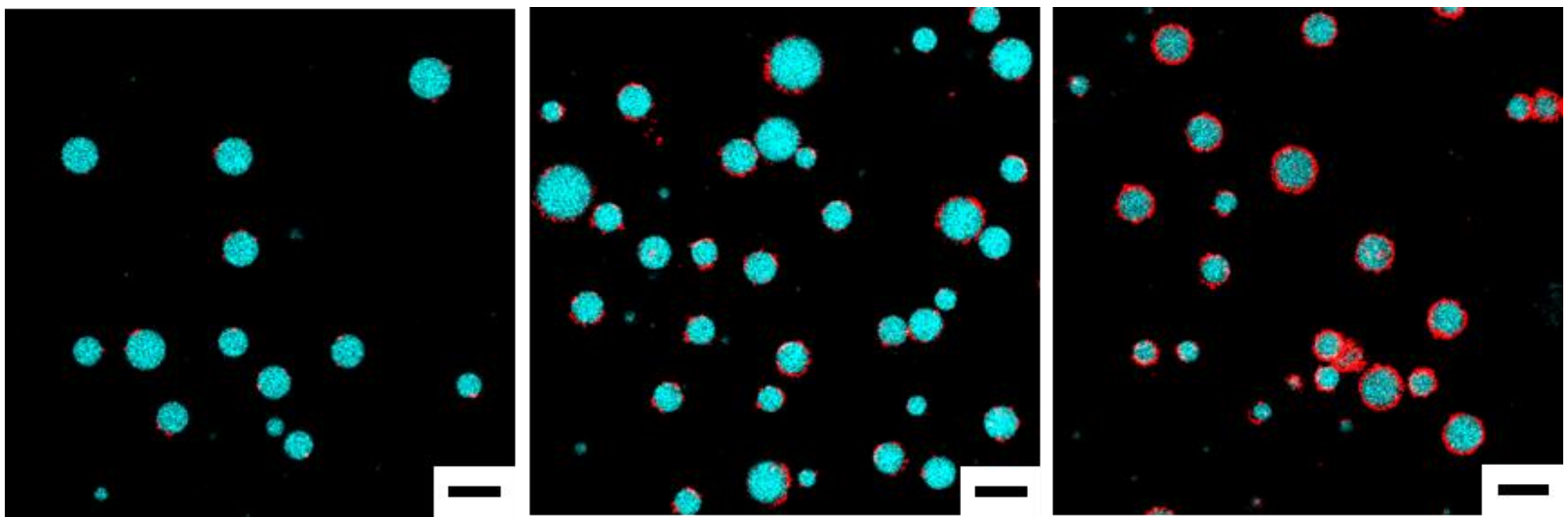

Figure S2. CLSM images of stomatocytes inserted into the membrane of terpolymer-stabilized amylose-based coacervates. Composition is 33 µL 1 mg/mL Cm-Am solution, 4.76 mg/mL stomatocyte solution, and 66 µL 1 mg/mL Q-Am solution, which are mixed by shaking for 2 min, after which terpolymer (5 µL, 25 mg/mL in PEG 350) is added. From left to right, the amount of stomatocyte solution added to the system is 1, 5, and 10 µL, respectively. Amylose-based coacervates are shown in cyan, stomatocytes are loaded with Cy5.5 (red). Scale bars represent 10 µm.

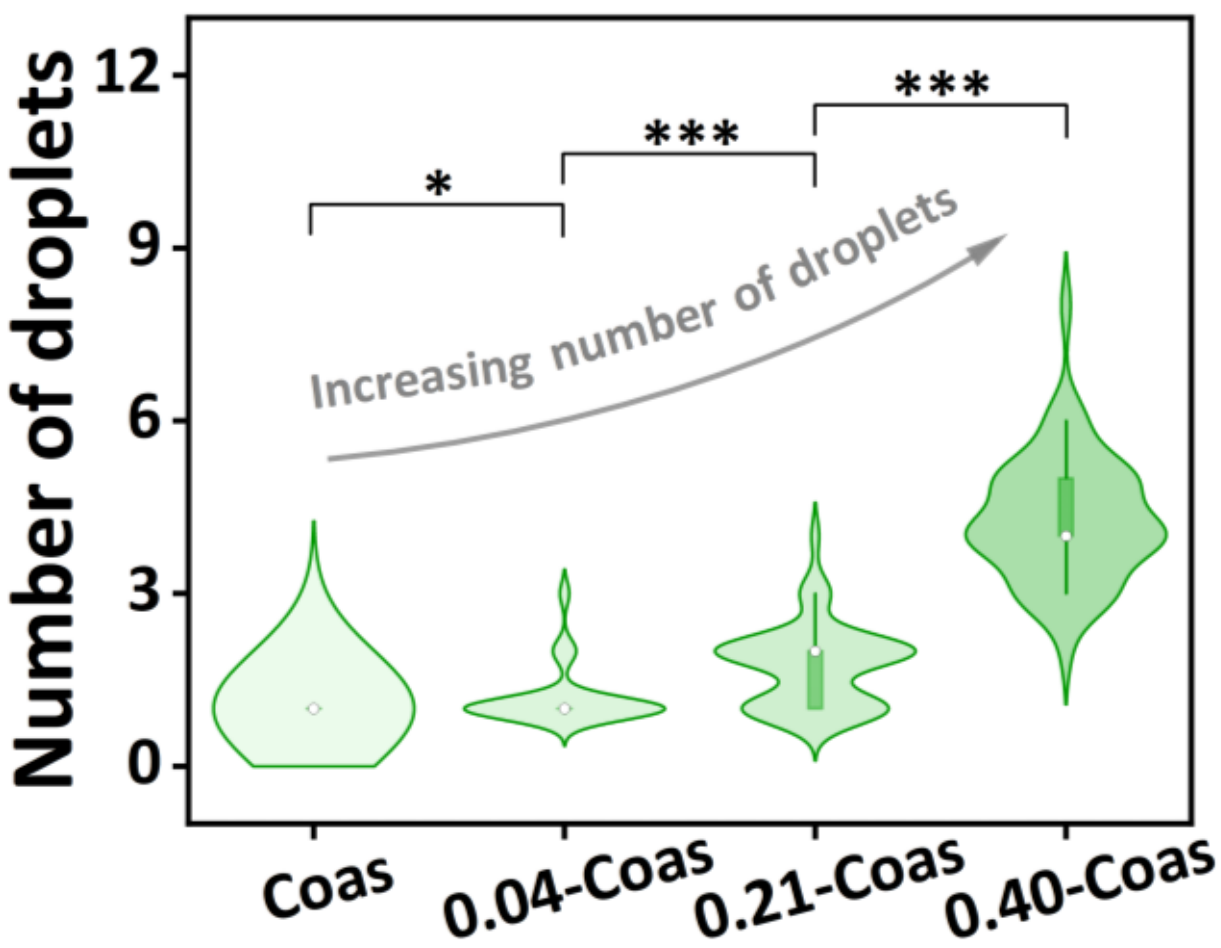

Figure S3. Quantification of the number of BSA-enriched subdroplets within individual coacervates. The analysis shown here reflects a coarse estimation based solely on features resolved in the x–y plane and does not represent the complete number of internal subdroplets present throughout the full 3D volume of each coacervate. For each condition, n=50 coacervates were measured. Statistical significance between conditions is indicated by the asterisk (*$p < 0.05$, **$p < 0.01$, ***$p < 0.001$). In all violin plots, the dark green box represents the interquartile range (IQR), the dark green line indicates 1.5 × IQR, and the white circle represents the median.

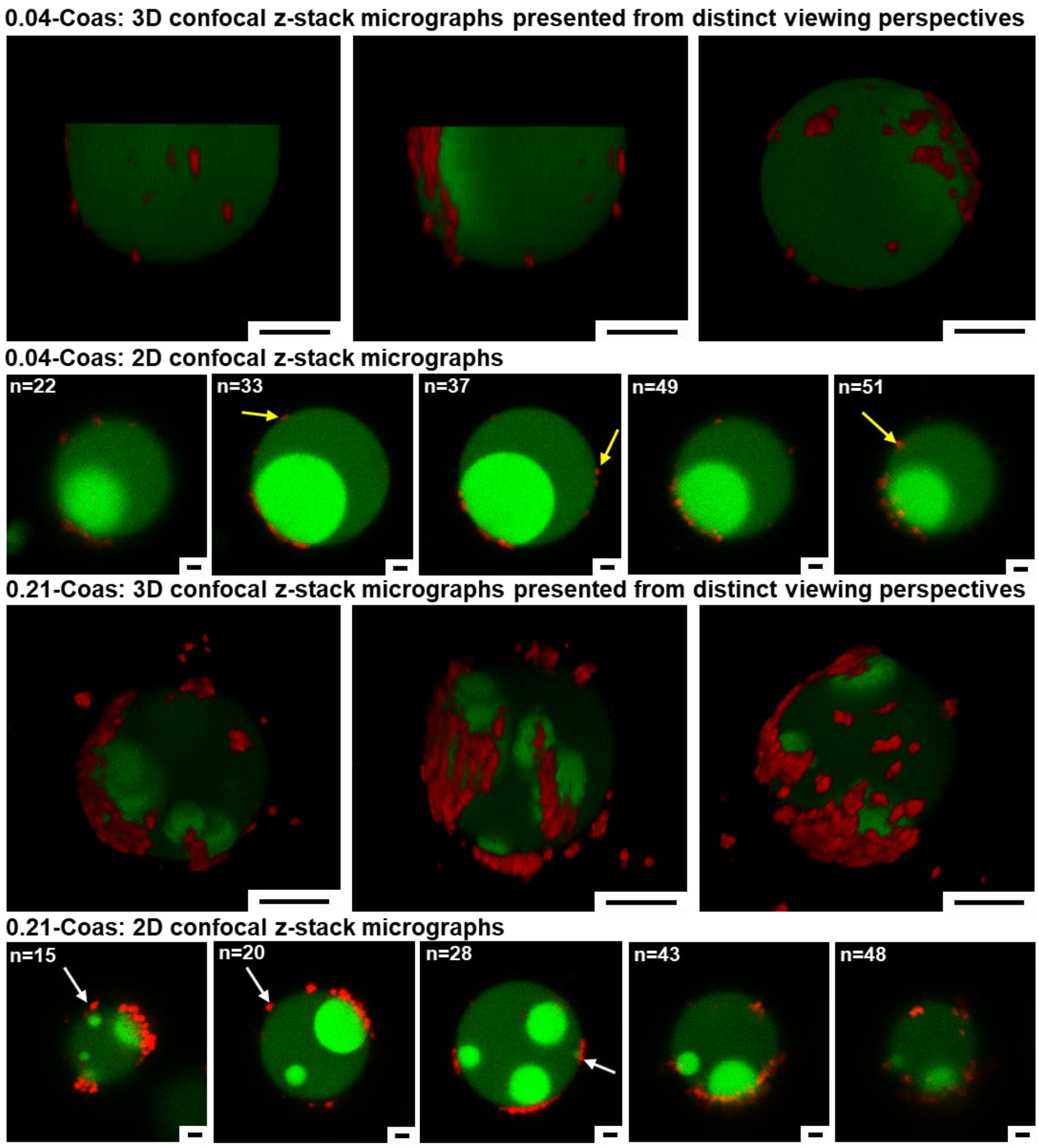

Figure S4. 2D and 3D z-stack micrographs of representative 0.04-Coas and 0.21-Coas droplets. The arrow points to stomatocytes scattered at the interface of coacervates. For each droplet, a total of 60 optical slices were acquired from the bottom to the top (or vice versa). The number *n* denotes the *n*-th optical slice counted from the starting plane (bottom or top) of the droplet. The 0.04-Coas and 0.21-Coas shown here correspond to the same droplets as those in Figure 2, imaged from different viewing angles. Red: stomatocytes loaded with Cy5.5, green: ATTO-488 conjugated suc-BSA. Scale bars represent 1 µm (2D micrographs) and 5 µm (3D micrographs).

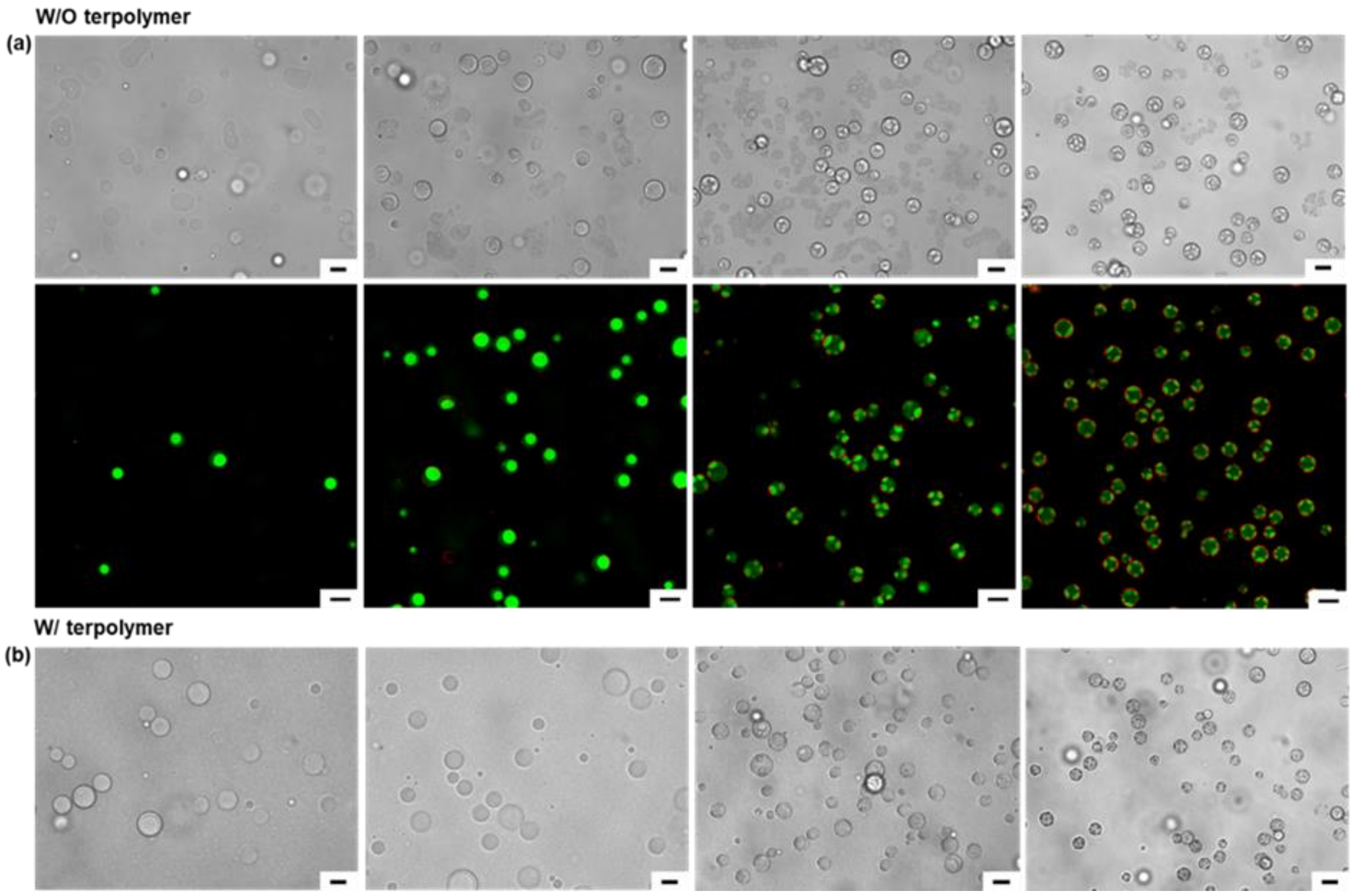

Figure S5. CLSM images of stomatocyte-stabilized multiphase coacervates (a) and stomatocyte insert into terpolymer-stabilized multiphase coacervates (b). (a) Samples were prepared by mixing 33 µL of Cm–Am solution (1 mg/mL); 0, 1, 5, or 10 µL of stomatocyte dispersion (4.76 mg/mL; left to right); 5 µL of suc-BSA solution (3.33 mg/mL); and 66 µL of Q–Am solution (1 mg/mL), followed by shaking for 4 min. (b) Samples were prepared as in (a), after which terpolymer (5 µL, 25 mg/mL in PEG 350) was added. Stomatocytes were labeled with Cy5.5 (red), and suc-BSA was conjugated with ATTO-488 (green). Scale bars: 10 µm.

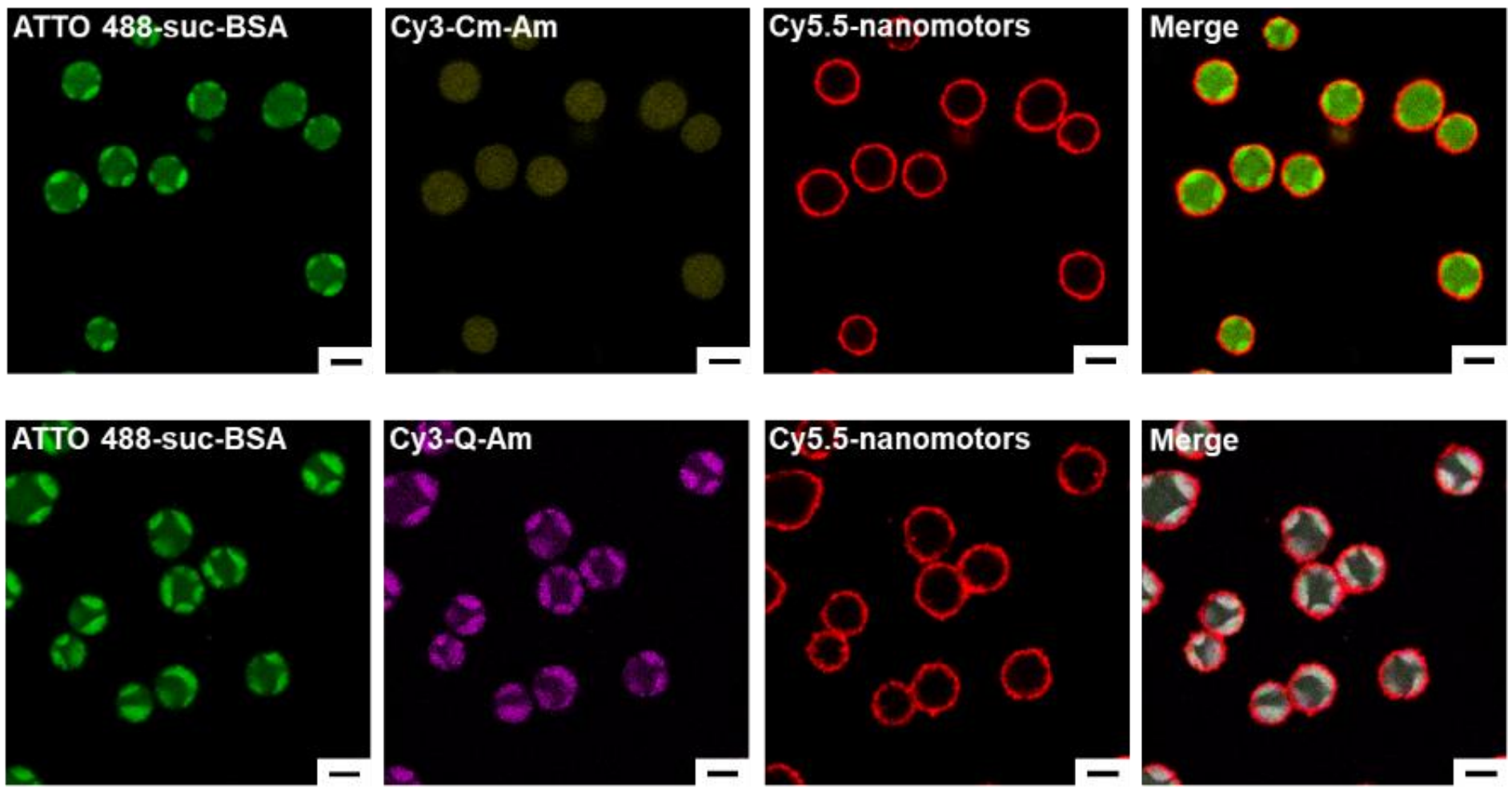

Figure S6. CLSM figures of multiphase-separated stomatocyte-stabilized coacervates (in the absence of terpolymer). Composition is 33 μL 1 mg/mL Cm-Am solution (containing 2 μL 1 mg/mL Cy3-Cm-Am), 20 μL 4.76 mg/mL stomatocyte solution, 5 μL 3.33 mg/mL suc-BSA solution, and 66 μL 1 mg/mL Q-Am (containing 2 μL 1 mg/mL Cy3-Q-Am) solution, which are mixed by shaking for 4 min. Red: stomatocytes loaded with Cy5.5, green: ATTO-488 conjugated suc-BSA, yellow: Cy3 conjugated Cm-Am, magenta: Cy3 conjugated Q-Am. Scale bars represent 5 μm.

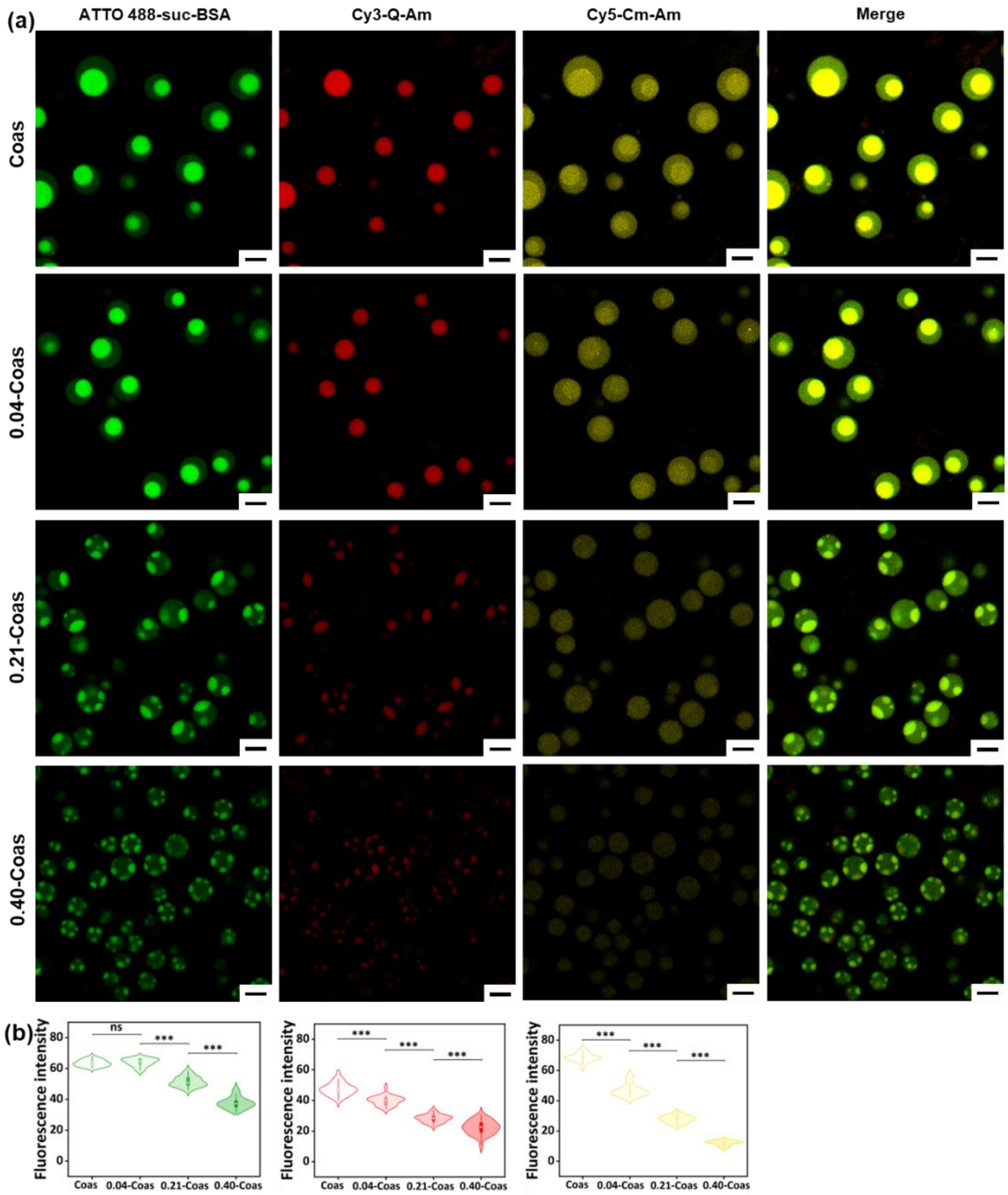

Figure S7. (a) CLSM figures of Coas, 0.04-Coas, 0.21-Coas, and 0.40-Coas. Red: Cy3 conjugated Q-Am, yellow: Cy5 conjugated Cm-Am, green: ATTO-488 conjugated suc-BSA. Stomatocytes were not dye-labeled. Scale bars represent 5 µm. (b) Quantification of fluorescence intensity of suc-BSA (left), Q-Am (middle), and Cm-Am (right) within Coas, 0.04-Coas, 0.21-Coas, and 0.40-Coas. For each condition, n=30 coacervates were measured. Statistical significance between conditions is indicated by the asterisk (***$p < 0.001$). In all violin plots, the box represents the interquartile range (IQR), the dark line indicates 1.5 × IQR, and the white circle represents the median.

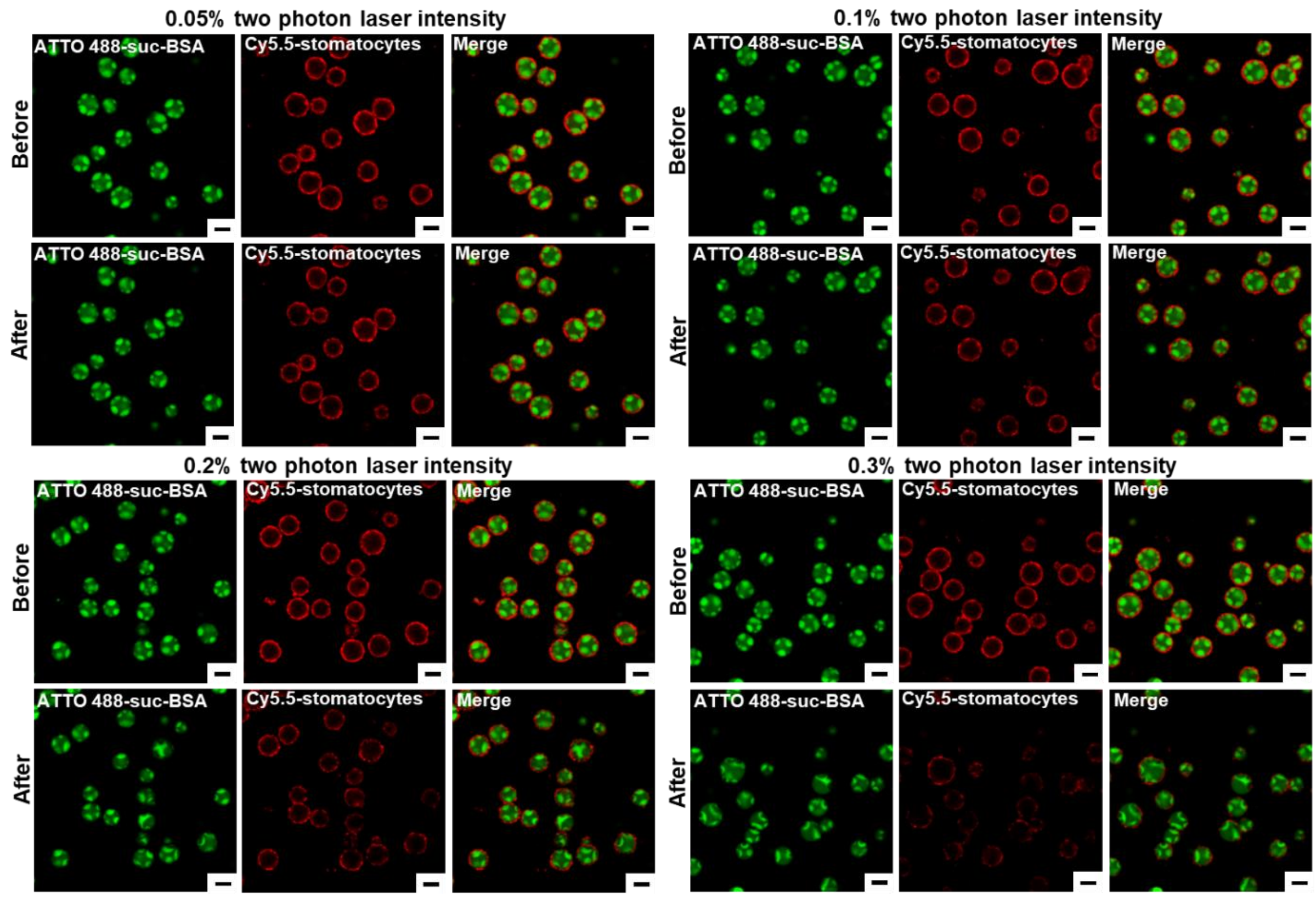

Figure S8. CLSM images of multiphase-separated 0.40-Coas before two-photon (TP) laser irradiation and after 1-min TP laser irradiation. The TP laser intensity is 0.05%, 0.1%, 0.2%, and 0.3%, respectively. Red: stomatocytes loaded with Cy5.5, green: ATTO-488 conjugated suc-BSA. Scale bars represent 5 μm.

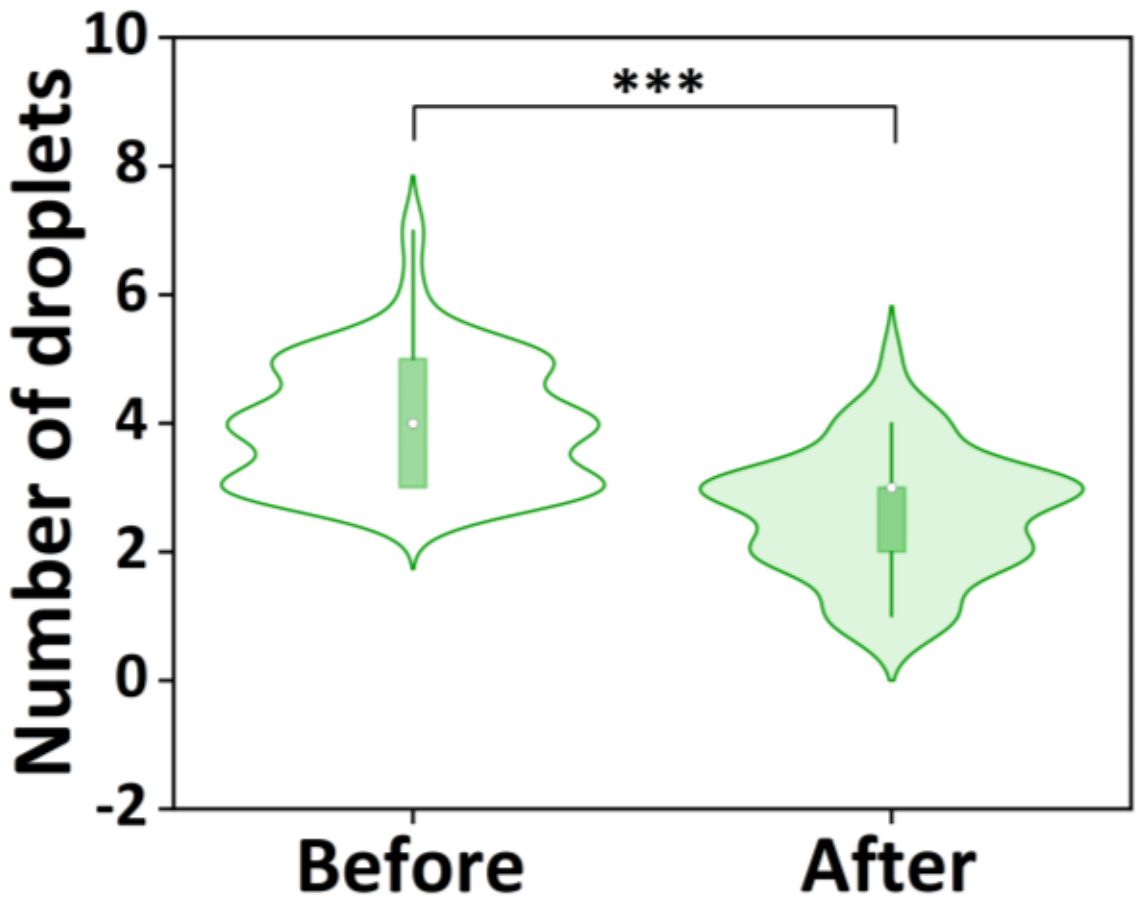

Figure S9. Quantification of the number of BSA-enriched subdroplets within 0.40-Coas before TP laser irradiation and after 1-min 0.3% TP laser irradiation. The analysis shown here reflects a coarse estimation based on features resolved in the x–y plane and does not represent the complete number of internal subdroplets present throughout the full 3D volume of each coacervate. 50 coacervates were measured. Statistical significance between conditions is indicated by the asterisk (***$p < 0.001$). In all violin plots, the dark green box represents the interquartile range (IQR), the dark green line indicates 1.5 × IQR, and the white circle represents the median.

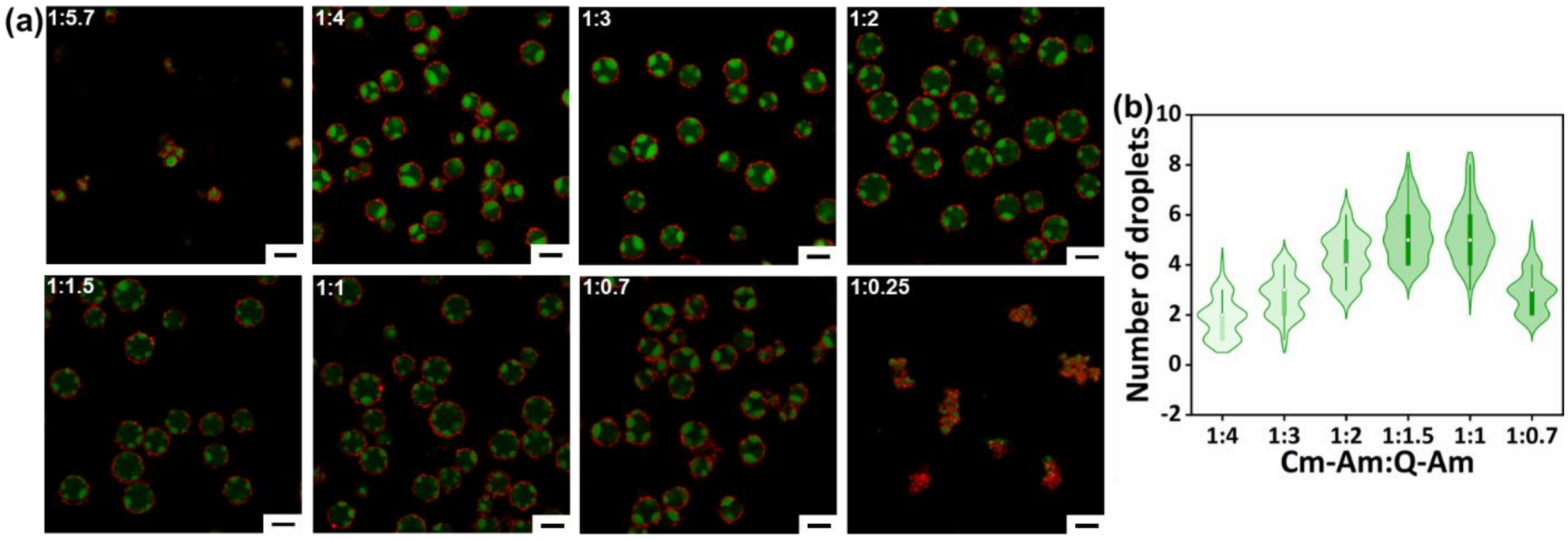

Figure S10. CLSM images of multiphase-separated 0.40-Coas formed at different volume ratios of Cm-Am to Q-Am (1:5.7-1:0.25). The total volume of the amylose components was 100 µL. The composition includes 1 mg/mL Cm-Am solution, 10 µL of 4.76 mg/mL stomatocyte solution, 5 µL of 3.33 mg/mL suc-BSA solution, and 1 mg/mL Q-Am solution, which were mixed by shaking for 4 minutes, followed by the addition of the terpolymer. Red: stomatocytes labeled with Cy5.5; Green: ATTO-488 conjugated suc-BSA. Scale bars represent 5 µm. (b) Quantification of the number of BSA-enriched subdroplets within the 0.40-Coas formed at different Cm-Am to Q-Am volume ratios.

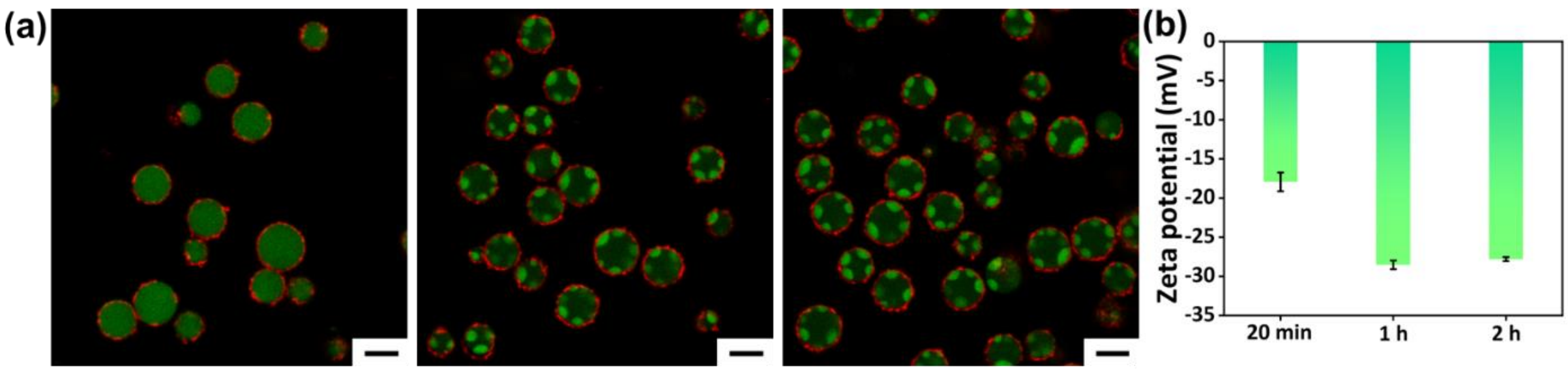

Figure S11. (a) CLSM images of multiphase-separated 0.40-Coas formed using suc-BSA with different charge densities. The succinylation times of BSA were 20 min, 1 h, and 2 h (from left to right). Red: stomatocytes loaded with Cy5.5; green: ATTO 488–conjugated suc-BSA. Scale bars: 5 µm. (b) Zeta potentials of suc-BSA prepared with different succinylation time.

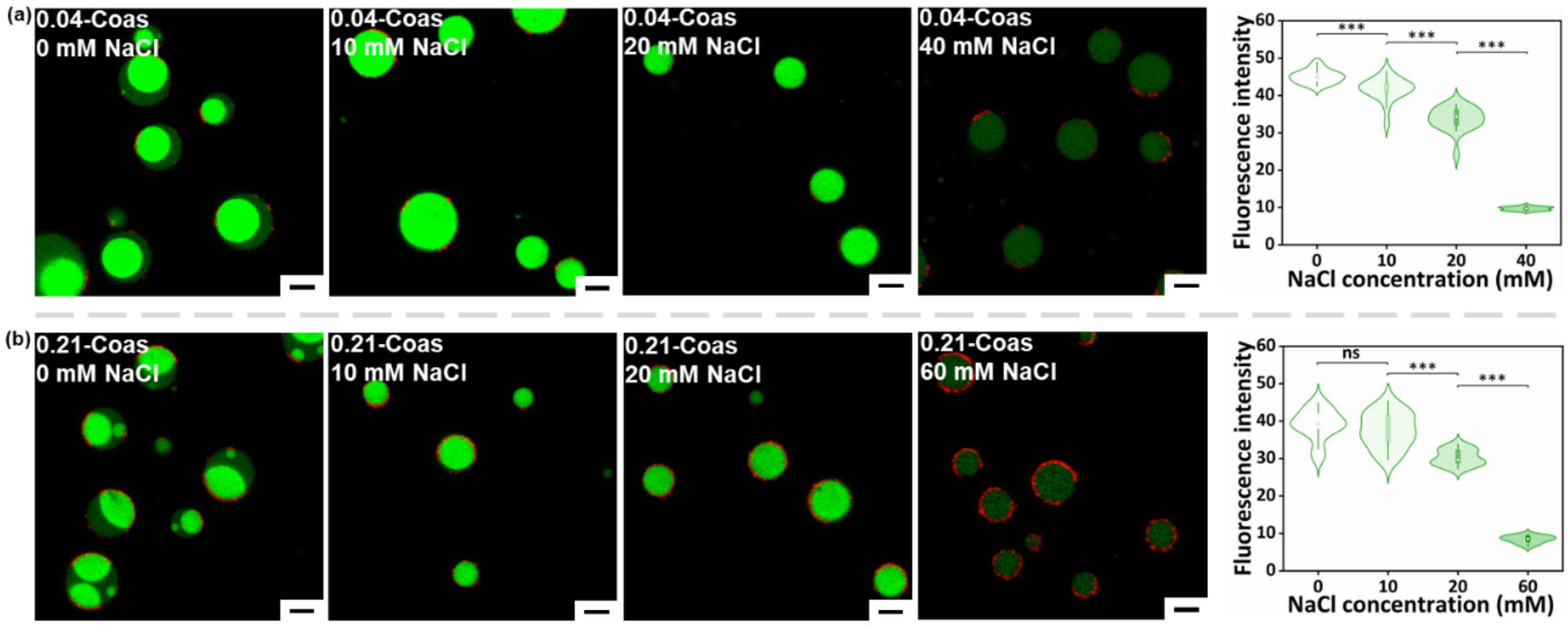

Figure S12. CLSM images (left) illustrating NaCl-induced evolution of the multiphase architecture in 0.04-Coas (a) and 0.21-Coas (b). Corresponding fluorescence intensity quantification (right) of BSA-enriched droplets in 0.04-Coas (a) and 0.21-Coas (b) as a function of NaCl concentration. Upon homogeneous distribution of BSA within the coacervates, the fluorescence signal was no longer localized to discrete BSA-enriched droplets. Red: Cy5.5-labeled stomatocytes, green: ATTO-488 conjugated suc-BSA. Scale bars: 5 µm. Images in panel (a) and (b) were each processed using separate, internally consistent brightness and contrast settings. All images were shown for visualization purposes only. Quantitative fluorescence analysis was performed using raw, unprocessed images. Statistical significance between conditions is indicated by the asterisk (***$p < 0.001$). In all violin plots, the green box represents the interquartile range (IQR), the green line indicates 1.5 × IQR, and the white circle represents the median.

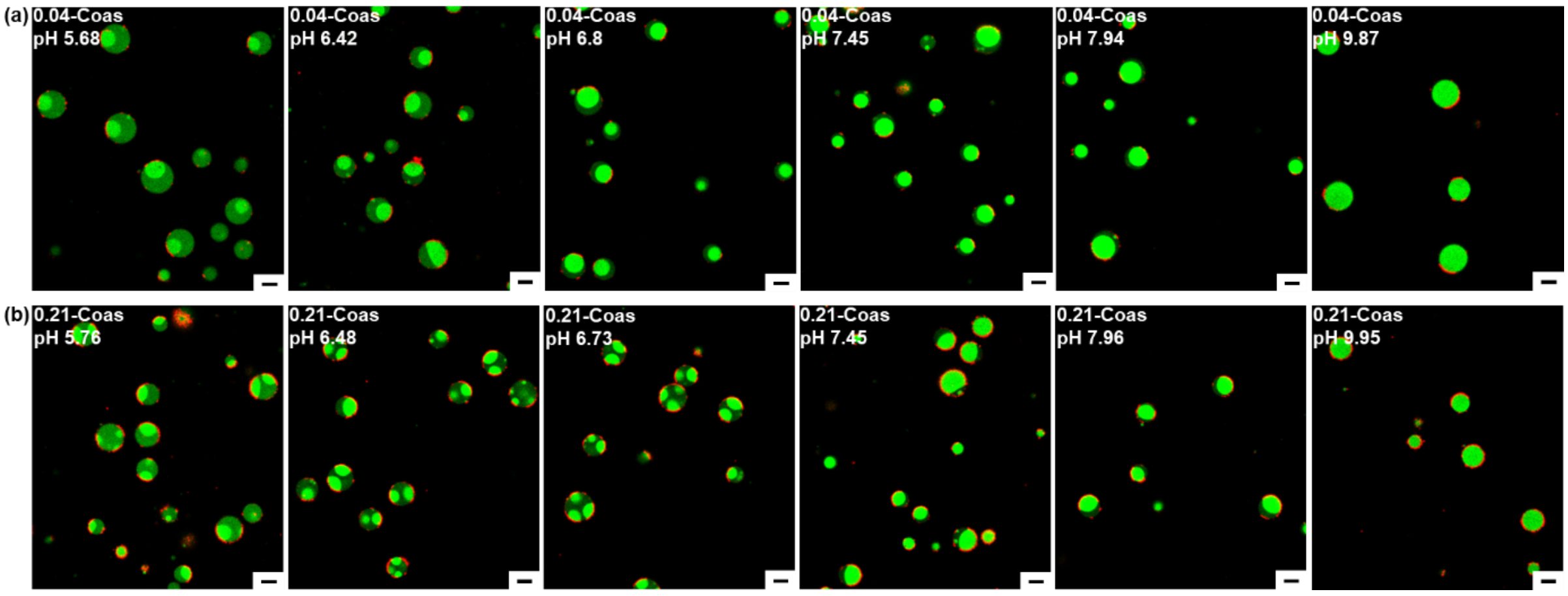

Figure S13. CLSM images illustrating pH-induced evolution of the multiphase architecture in 0.04-Coas (a) and 0.21-Coas (b). Red: Cy5.5-labeled stomatocytes; green: ATTO-488 conjugated suc-BSA. Scale bars: 5 µm. Images in panel (a) and (b) were each processed using separate, internally consistent brightness and contrast settings. All images were shown for visualization purposes only.

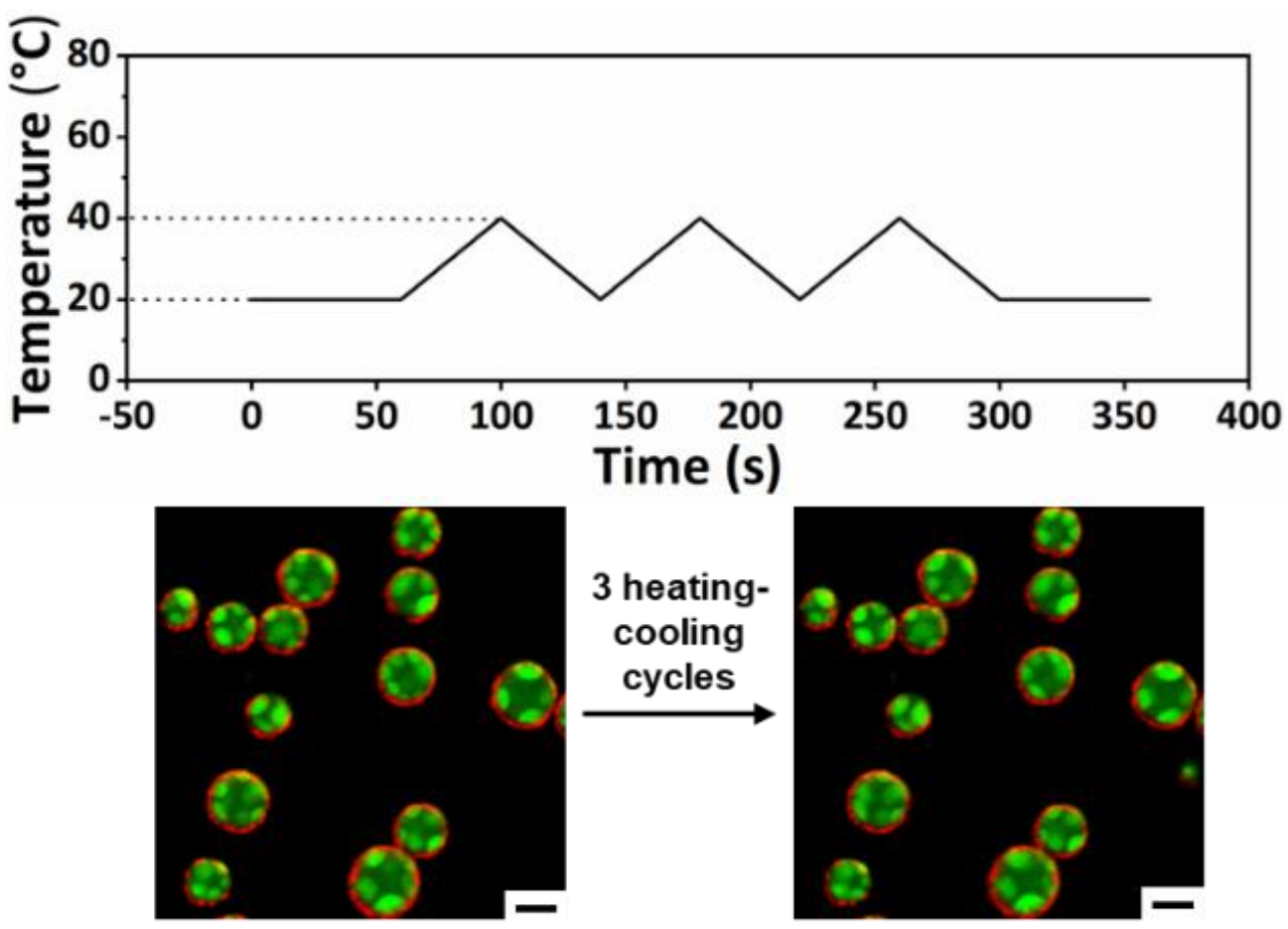

Figure S14. CLSM images showing temperature-induced changes in the multiphase structure of 0.40-Coas after three heating–cooling cycles between 20 and 40 °C (heating and cooling rates: 30 °C /min). Red: Cy5.5-labeled stomatocytes; green: ATTO-488 conjugated suc-BSA. Scale bars: 5 µm. Images were processed with identical brightness and contrast settings and were shown solely for visualization; they were not used for quantitative fluorescence analysis.

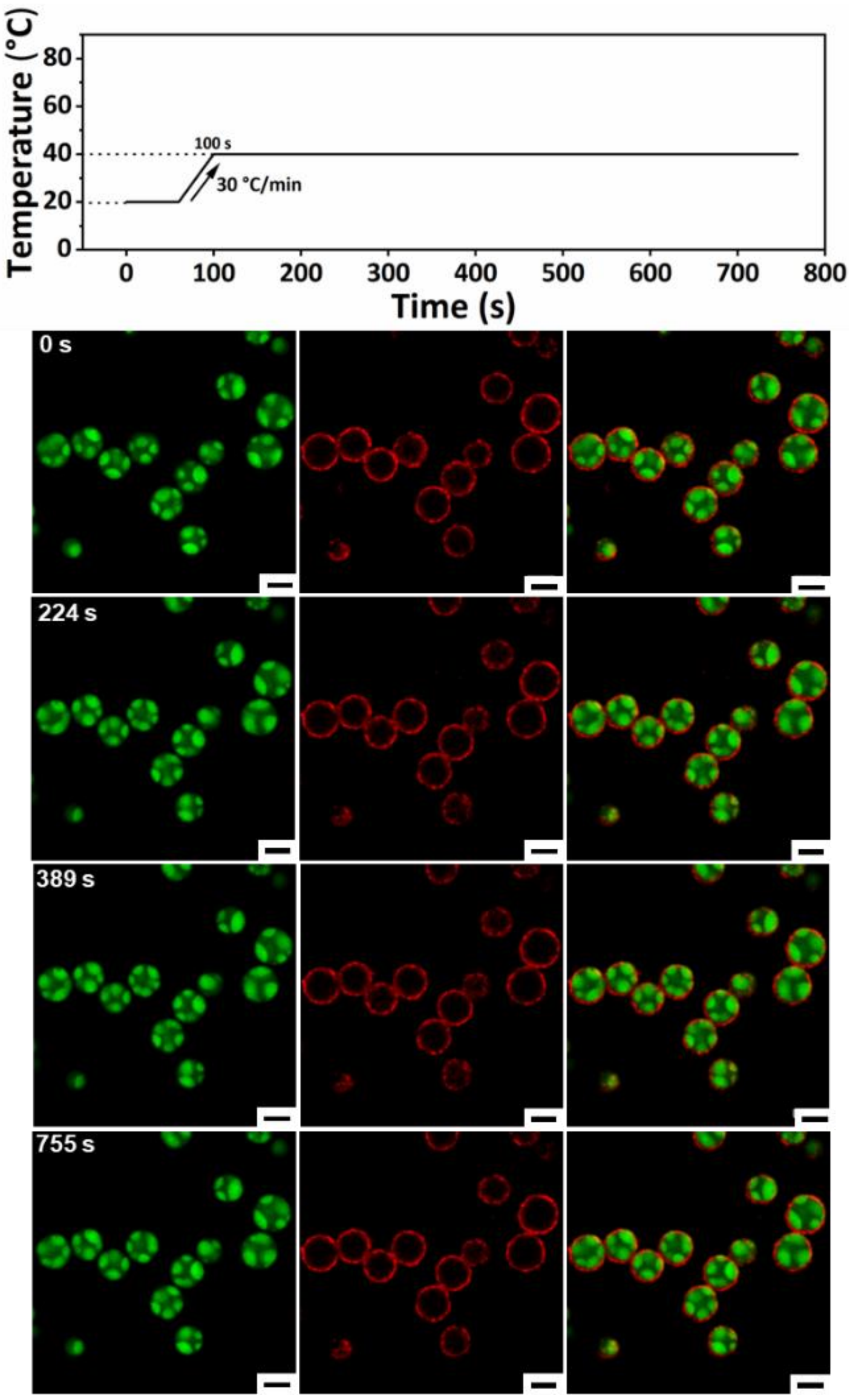

Figure S15. CLSM images showing temperature-induced changes in the multiphase structure of 0.40-Coas upon heating from 20 to 40 °C at 30 °C /min with a hold time >10 min. Red: Cy5.5-labeled stomatocytes; green: ATTO-488 conjugated suc-BSA. Scale bars: 5 µm. Images were processed with identical brightness and contrast settings and were shown solely for visualization; they were not used for quantitative fluorescence analysis.

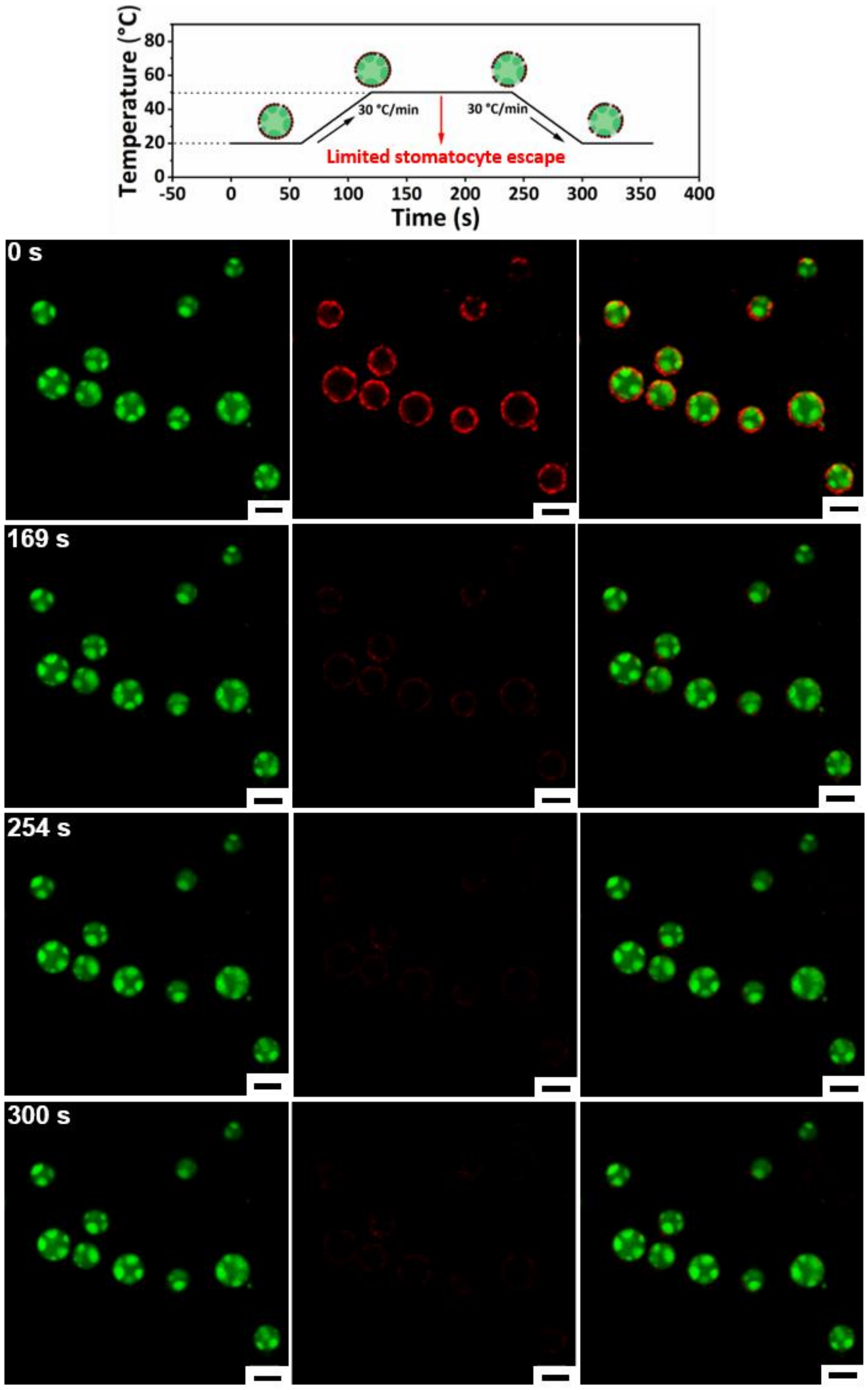

Figure S16. CLSM images showing temperature-induced changes in the multiphase structure of 0.40-Coas upon heating from 20 to 50 °C (2 min hold) followed by cooling to 20 °C. Heating and cooling rates were 30 °C /min. Red: Cy5.5-labeled stomatocytes; green: ATTO-488 conjugated suc-BSA. Scale bars: 5 µm. Images were processed with identical brightness and contrast settings and were shown solely for visualization; they were not used for quantitative fluorescence analysis.

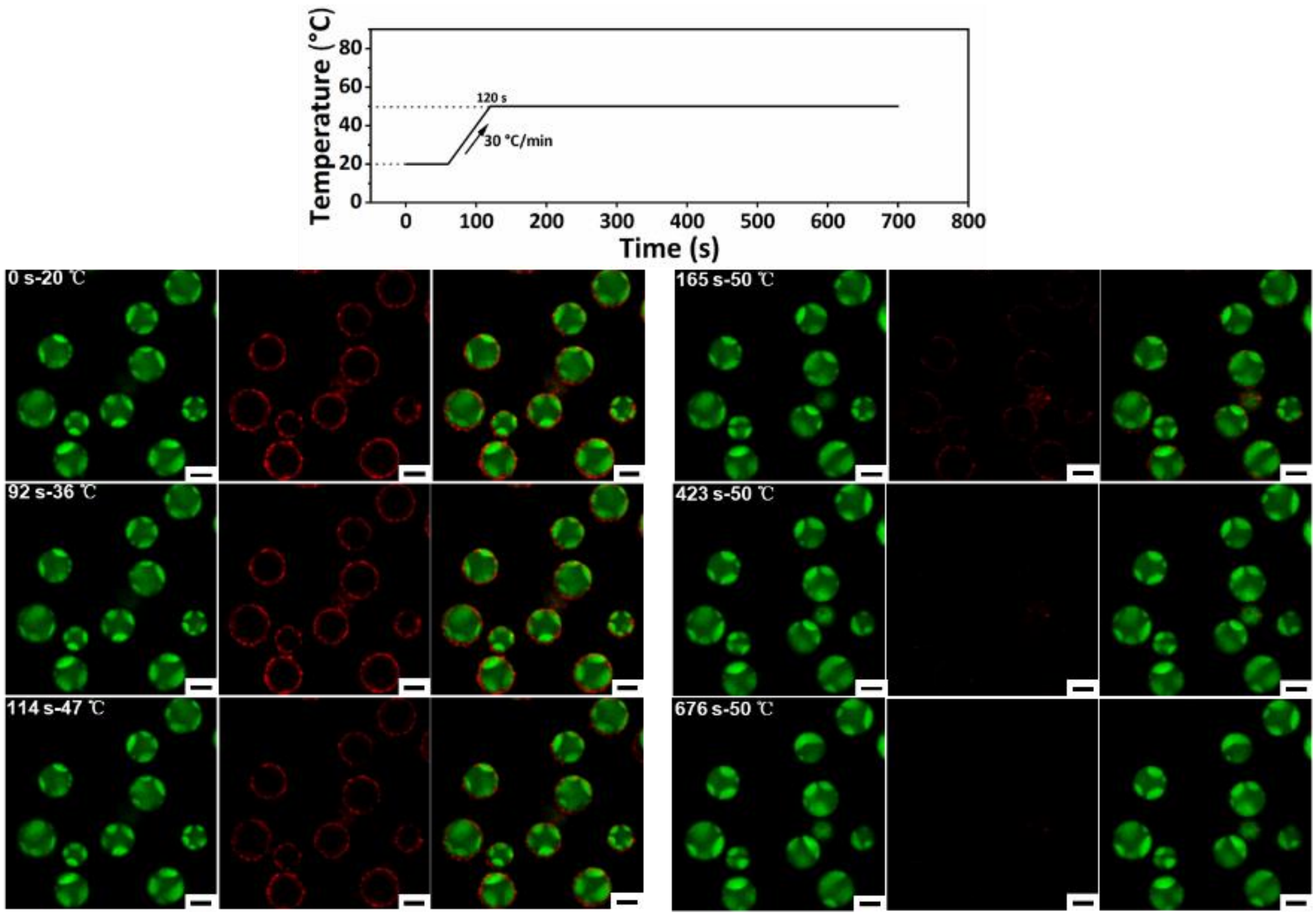

Figure S17. CLSM images showing temperature-induced changes in the multiphase structure of 0.40-Coas upon heating from 20 to 50 °C at 30 °C /min with a hold time >10 min. Red: Cy5.5-labeled stomatocytes; green: ATTO-488 conjugated suc-BSA. Scale bars: 5 µm. Images were processed with identical brightness and contrast settings and were shown solely for visualization; they were not used for quantitative fluorescence analysis.

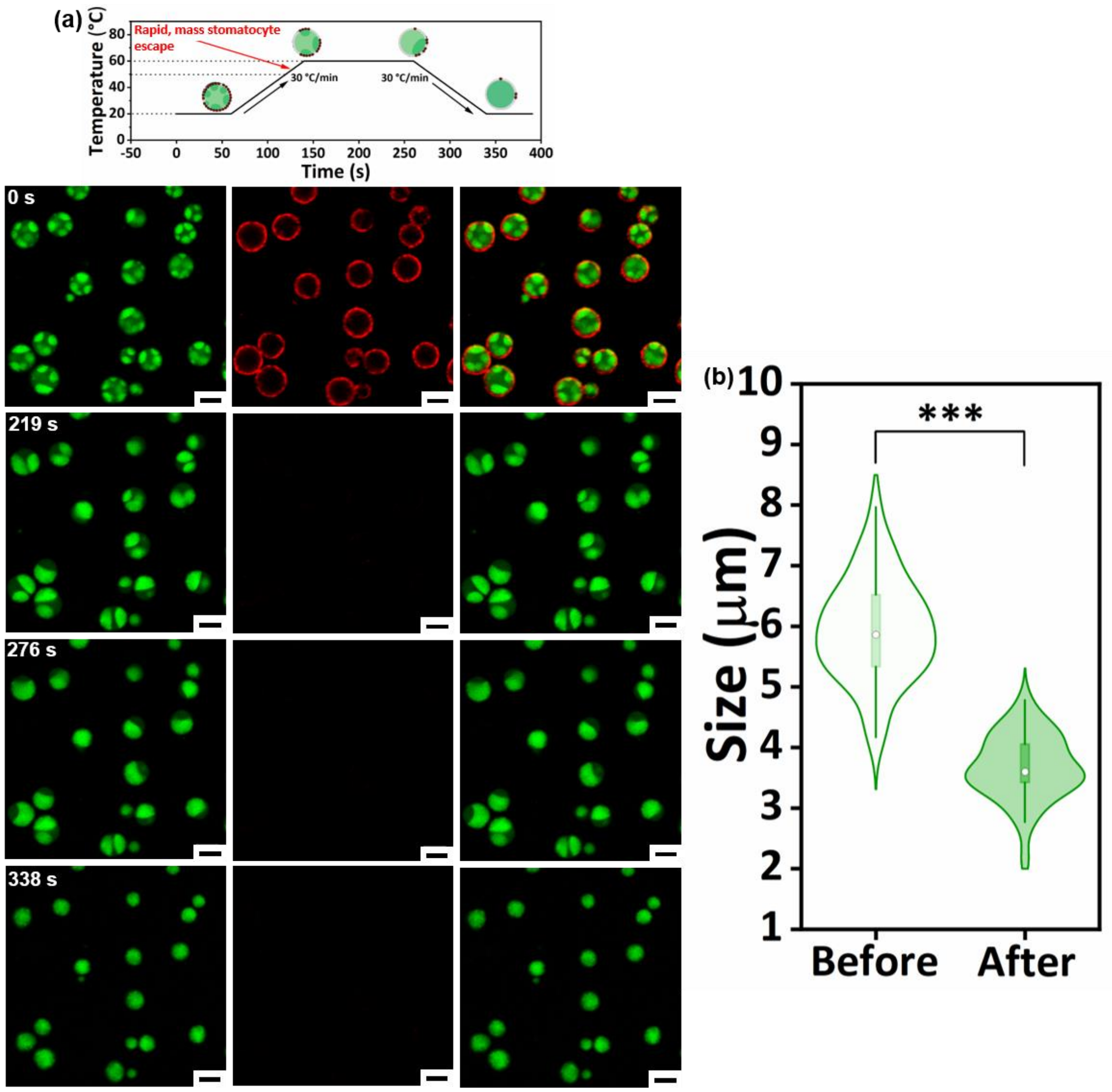

Figure S18. (a) CLSM images showing temperature-induced changes in the multiphase structure of 0.40-Coas upon heating from 20 to 60 °C (2 min hold) followed by cooling to 20 °C. Heating and cooling rates were 30 °C /min. Red: Cy5.5-labeled stomatocytes; green: ATTO-488 conjugated suc-BSA. Scale bars: 5 µm. Images were processed with identical brightness and contrast settings and were shown solely for visualization; they were not used for quantitative fluorescence analysis. (b) Size analysis of coacervates before stomatocyte escape and after stomatocyte escape. 38 coacervates were analyzed for each condition.

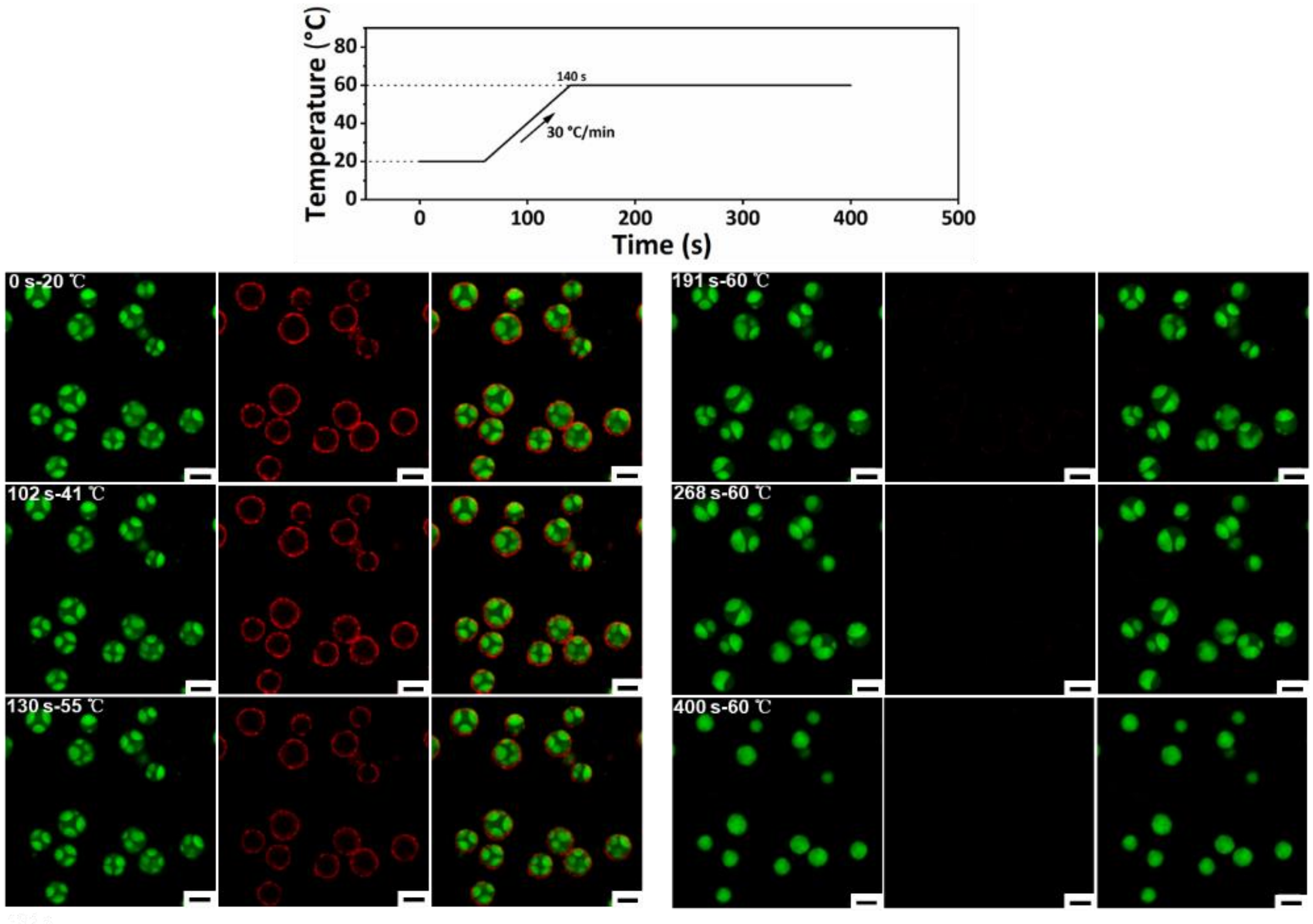

Figure S19. CLSM images showing temperature-induced changes in the multiphase structure of 0.40-Coas upon heating from 20 to 60 °C at 30 °C /min with a hold time >10 min. Red: Cy5.5-labeled stomatocytes; green: ATTO-488 conjugated suc-BSA. Scale bars: 5 µm. Images were processed with identical brightness and contrast settings and were shown solely for visualization; they were not used for quantitative fluorescence analysis.

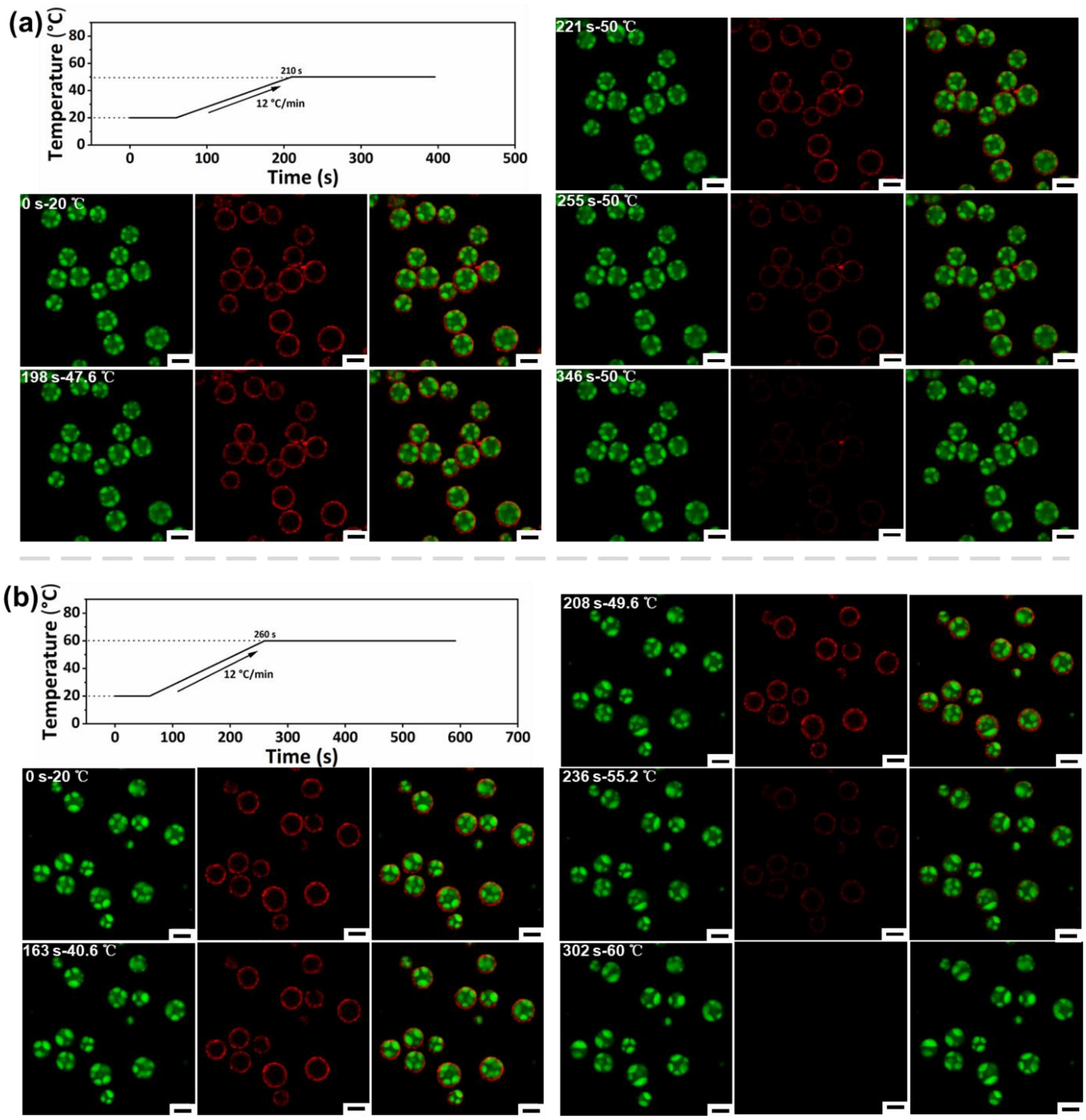

Figure S20. CLSM images showing temperature-induced changes in the multiphase structure of 0.40-Coas upon heating from 20 to 50 °C at 12 °C /min with a hold time >10 min (**a**), and upon heating from 20 to 60 °C at 12 °C /min with a hold time >10 min (**b**). Red: Cy5.5-labeled stomatocytes; green: ATTO-488 conjugated suc-BSA. Scale bars: 5 µm. Images were processed with identical brightness and contrast settings and were shown solely for visualization; they were not used for quantitative fluorescence analysis.

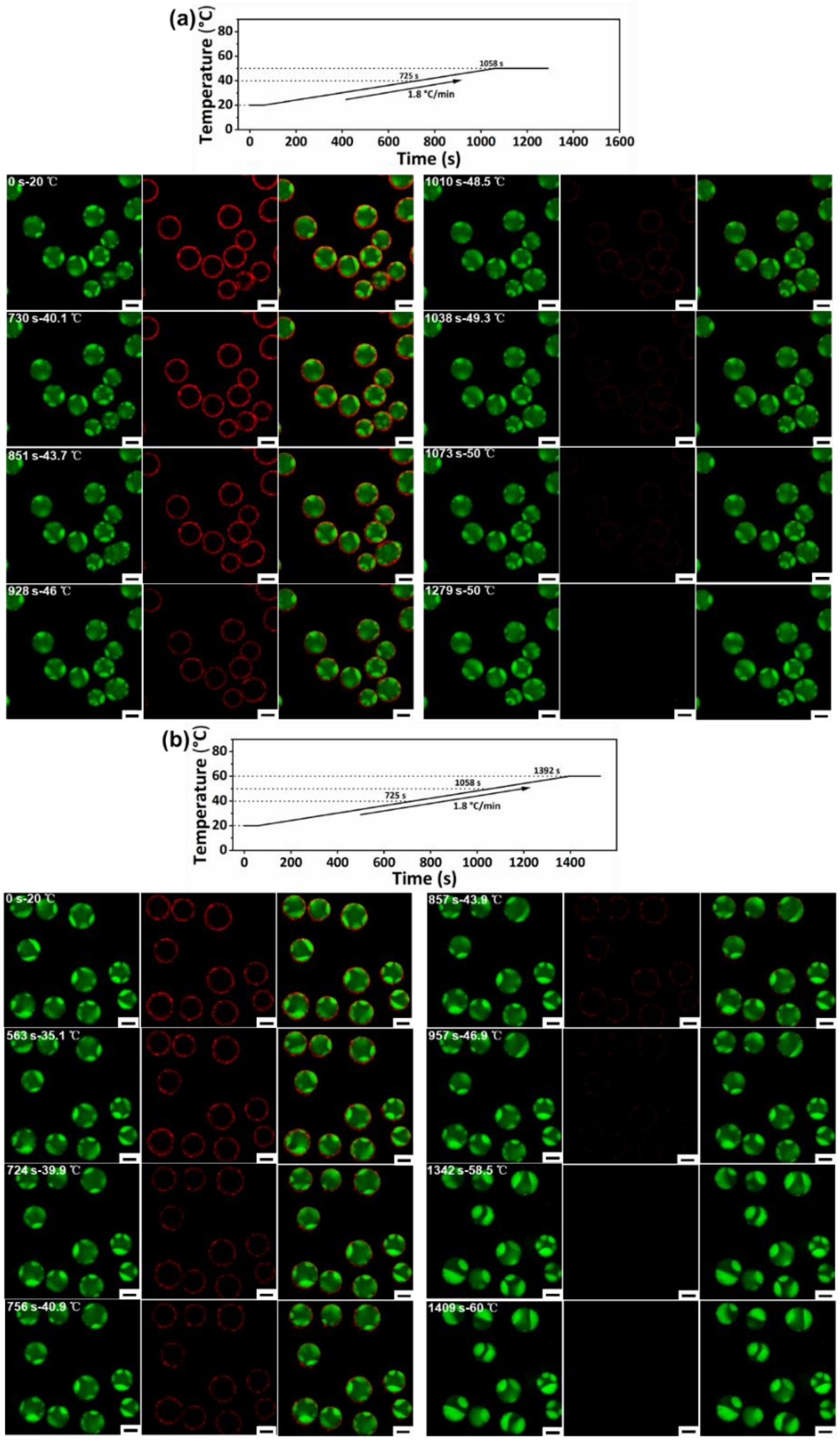

Figure S21. CLSM images showing temperature-induced changes in the multiphase structure of 0.40-Coas upon heating from 20 to 50 °C at 1.8 °C /min with a hold time >10 min (**a**), and upon heating from 20 to 60 °C at 1.8 °C /min with a hold time >10 min (**b**). Red: Cy5.5-labeled stomatocytes; green: ATTO-488 conjugated suc-BSA. Scale bars: 5 µm. Images were processed with identical brightness and contrast settings and were shown solely for visualization; they were not used for quantitative fluorescence analysis.

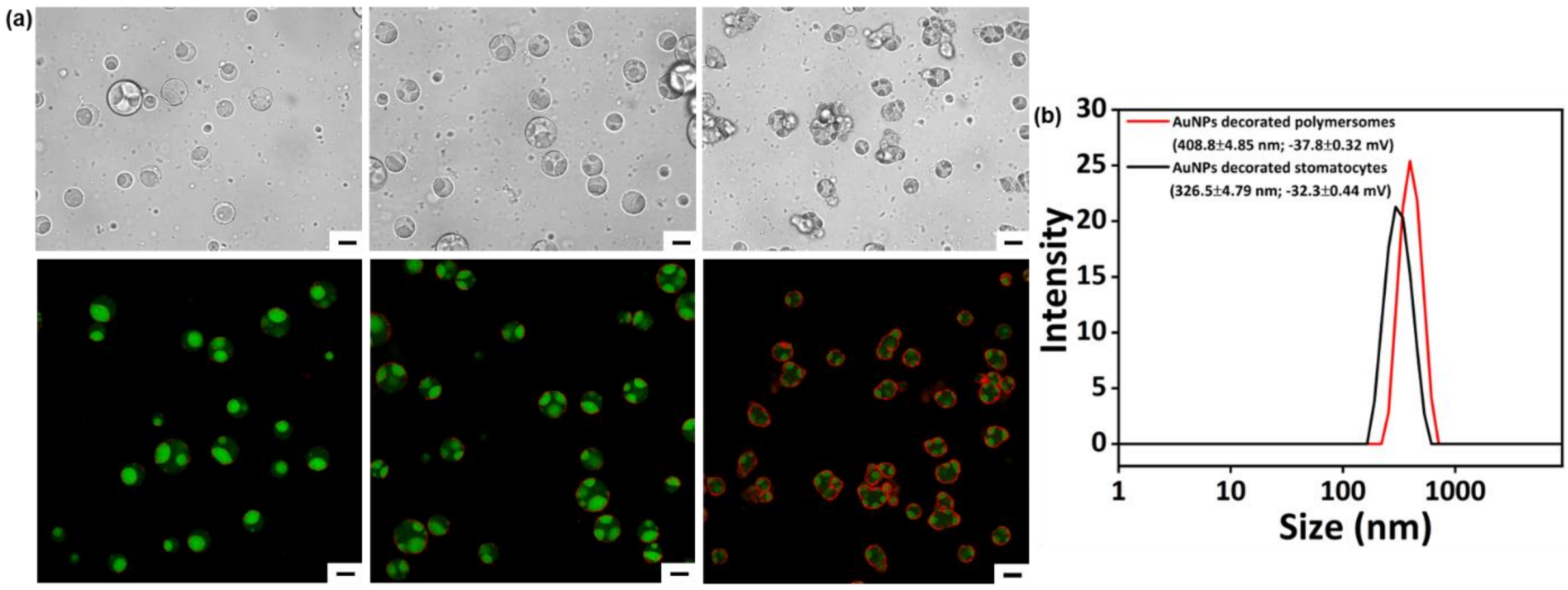

Figure S22. CLSM images of multiphase-separated polymersome-stabilized coacervates in the presence of terpolymer. Composition is 33 µL 1 mg/mL Cm-Am solution, 20 µL 4.76 mg/mL polymersome solution, 5 µL 3.33 mg/mL suc-BSA solution, and 66 µL 1 mg/mL Q-Am, which are mixed by shaking for 4 min. Subsequently, terpolymer (5 µL, 25 mg/mL in PEG 350) is added. From left to right, the amount of polymersome added is 1, 5, and 10 µL, respectively. suc-BSA is labeled with ATTO-488 (green), and polymersomes are labeled with Cy5.5 (red). Scale bars: 10 µm. (b) Size and zeta potential of AuNPs decorated polymersomes and stomatocytes.

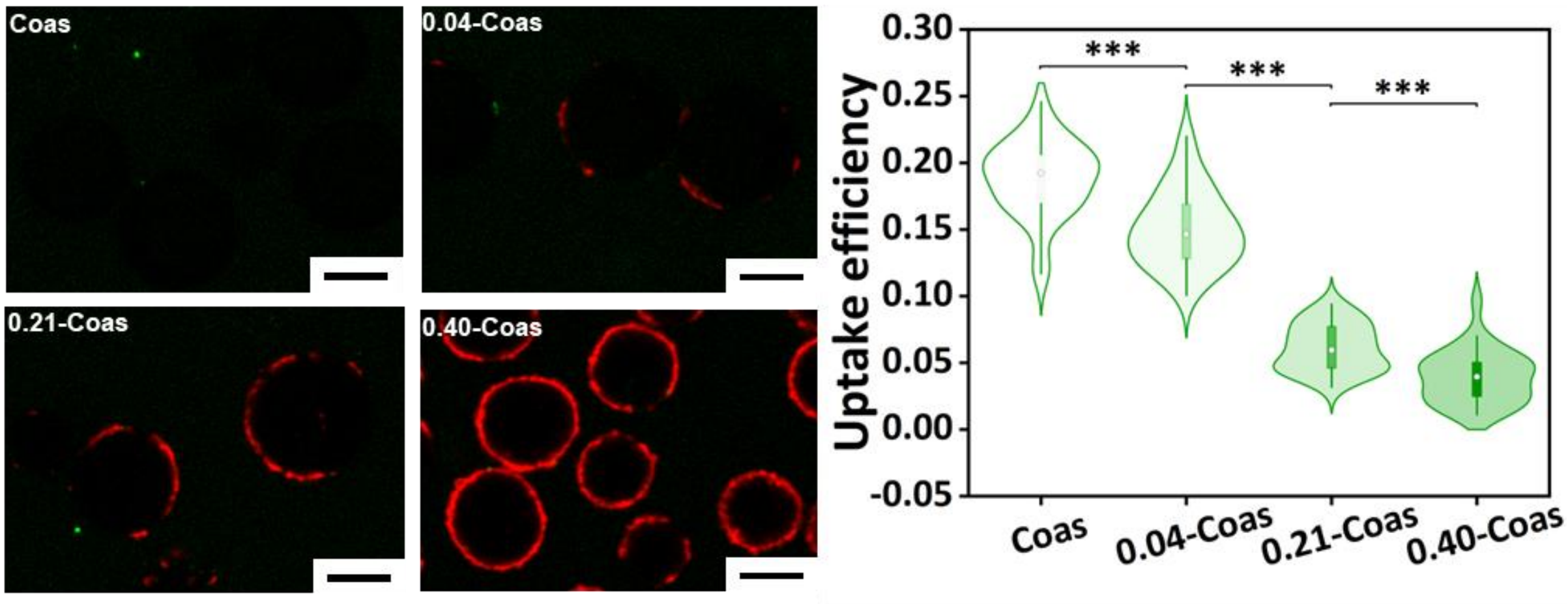

Figure S23. Uptake efficiency of Coas, 0.04-Coas, 0.21-Coas, and 0.40-Coas for neutral-charged GFP. For all experiments, n = 30 coacervates were measured. Statistical significance between conditions is indicated by the asterisk (***$p < 0.001$). In all violin plots, the green box represents the interquartile range (IQR), the green line indicates 1.5 × IQR, and the white circle represents the median. Permeability data were obtained from raw, unprocessed images. Scale bars: 5 μm. Green: GFP; red: Cy5.5-labeled stomatocytes.

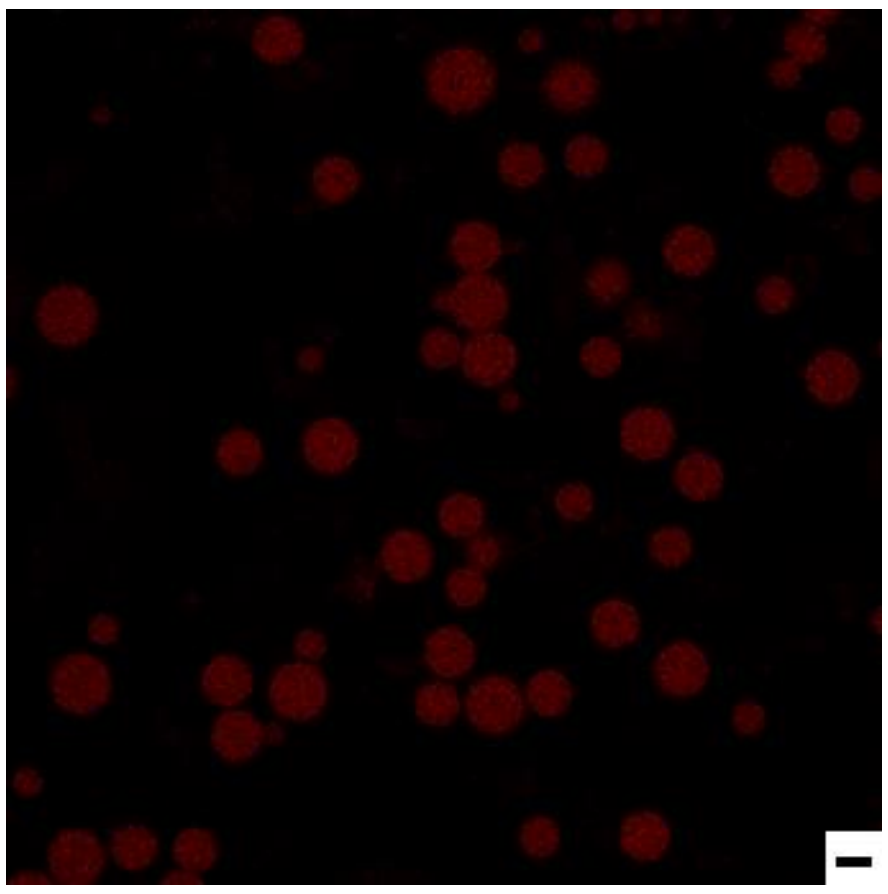

Figure S24. CLSM images of GOX-loaded terpolymer-stabilized amylose-based coacervates. Composition is 33 μL 1 mg/mL Cm-Am solution, 1 μL 2 mg/mL Cy3-GOX solution, and 66 μL 1 mg/mL Q-Am, which are mixed by shaking for 4 min. Subsequently, terpolymer (5 μL, 25 mg/mL in PEG 350) is added. Scale bars: 5 μm.

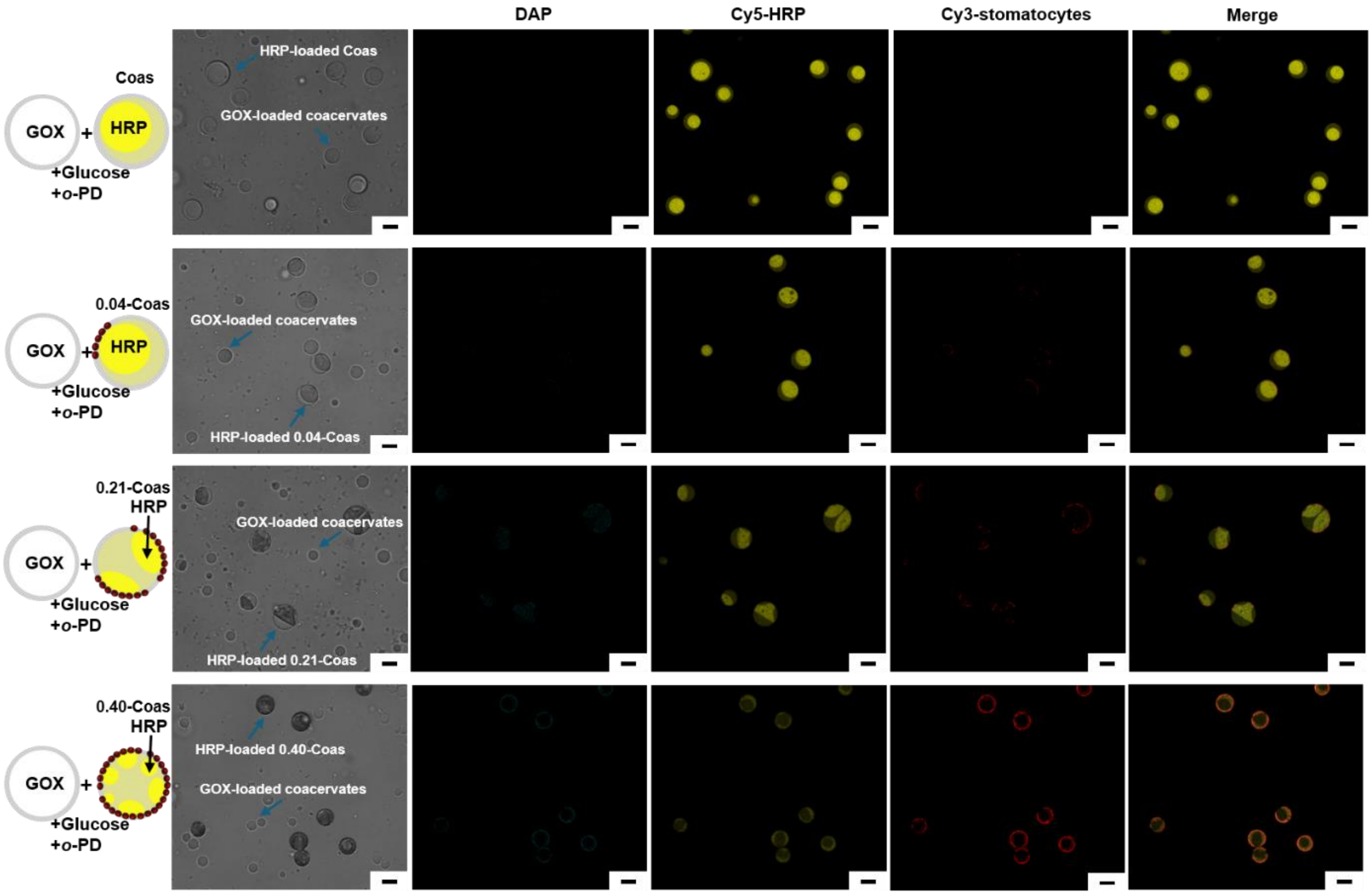

Figure S25. Stomatocyte-guided spatial organization regulates chemical communication between protocell populations. GOX encapsulated within terpolymer-stabilized amylose-based coacervates catalyzes the oxidation of glucose, generating H_2O_2 as a diffusible signaling molecule. The generated H_2O_2 is subsequently transferred to neighboring HRP-loaded coacervates (Coas, 0.04-Coas, 0.21-Coas, and 0.4-Coas), where HRP catalyzes the oxidation of *o*-PD into fluorescent DAP. 30 µL of GOX-loaded coacervates were mixed with 30 µL of HRP-loaded coacervates (Coas, 0.04-Coas, 0.21-Coas, and 0.4-Coas). Subsequently, 1 µL of 200 mM *o*-PD was added. Prior to video recording, 1 µL of 1 M glucose was introduced to initiate the enzymatic cascade reaction. CLSM images were recorded at 1 min after glucose addition. Scale bars: 10 µm.

References

1. Mason, A. F.; Altenburg, W. J.; Song, S.; van Stevendaal, M.; van Hest, J. C. M., Chapter Three - Terpolymer-Stabilized Complex Coacervates: A Robust and Versatile Synthetic Cell Platform. *Methods Enzymol.* **2021,** *646*, 51–82.

2. Mason, A. F.; Buddingh', B. C.; Williams, D. S.; van Hest, J. C. M., Hierarchical Self-Assembly of a Copolymer-Stabilized Coacervate Protocell. *J. Am. Chem. Soc.* **2017,** *139* (48), 17309-17312.

3. Novosedlik, S.; Cook, A. B.; Voermans, T. J. F. M.; Janssen, H. M.; van Hest, J. C. M., Control over Membrane Fluidity and Biophysical Properties of Synthetic Terpolymer Stabilized Complex Coacervates. *Polym. Chem.* **2024,** *15* (45), 4650-4661.

4. Pijpers, I. A. B.; Cao, S.; Llopis-Lorente, A.; Zhu, J.; Song, S.; Joosten, R. R. M.; Meng, F.; Friedrich, H.; Williams, D. S.; Sánchez, S.; van Hest, J. C. M.; Abdelmohsen, L. K. E. A., Hybrid Biodegradable Nanomotors through Compartmentalized Synthesis. *Nano Lett.* **2020,** *20* (6), 4472-4480.

5. Wang, J.; Wu, H.; Zhu, X.; Zwolsman, R.; Hofstraat, S. R. J.; Li, Y.; Luo, Y.; Joosten, R. R. M.; Friedrich, H.; Cao, S.; Abdelmohsen, L. K. E. A.; Shao, J.; van Hest, J. C. M., Ultrafast Light-Activated Polymeric Nanomotors. *Nat. Commun.* **2024,** *15* (1), 4878.

6. Erkamp, N. A.; Verwiel, M. A. M.; Qian, D.; Sneideris, T.; Spaepen, F. A.; Weitz, D. A.; van Hest, J. C. M.; Knowles, T. P. J., Biomolecular Condensates with Complex Architectures via Controlled Nucleation. *Nat. Chem. Eng.* **2024,** *1* (6), 430-439.
